# MRI of the Neonatal Brain: A Review of Methodological Challenges and Neuroscientific Advances

**DOI:** 10.1002/jmri.27192

**Published:** 2020-05-18

**Authors:** Jessica Dubois, Marianne Alison, Serena J. Counsell, Lucie Hertz‐Pannier, Petra S. Hüppi, Manon J.N.L. Benders

**Affiliations:** ^1^ University of Paris NeuroDiderot, INSERM,Paris France; ^2^ UNIACT, NeuroSpin, CEA; Paris‐Saclay University Gif‐sur‐Yvette France; ^3^ Department of Pediatric Radiology APHP, Robert‐Debré Hospital Paris France; ^4^ Centre for the Developing Brain School of Biomedical Engineering & Imaging Sciences, King's College London London UK; ^5^ Division of Development and Growth, Department of Woman, Child and Adolescent University Hospitals of Geneva Geneva Switzerland; ^6^ Department of Neonatology University Medical Center Utrecht, Utrecht University Utrecht the Netherlands

**Keywords:** brain development, newborns, infants, anatomical MRI, diffusion MRI, quantitative MRI, functional MRI

## Abstract

In recent years, exploration of the developing brain has become a major focus for researchers and clinicians in an attempt to understand what allows children to acquire amazing and unique abilities, as well as the impact of early disruptions (eg, prematurity, neonatal insults) that can lead to a wide range of neurodevelopmental disorders. Noninvasive neuroimaging methods such as MRI are essential to establish links between the brain and behavioral changes in newborns and infants. In this review article, we aim to highlight recent and representative studies using the various techniques available: anatomical MRI, quantitative MRI (relaxometry, diffusion MRI), multiparametric approaches, and functional MRI. Today, protocols use 1.5 or 3T MRI scanners, and specialized methodologies have been put in place for data acquisition and processing to address the methodological challenges specific to this population, such as sensitivity to motion. MR sequences must be adapted to the brains of newborns and infants to obtain relevant good soft‐tissue contrast, given the small size of the cerebral structures and the incomplete maturation of tissues. The use of age‐specific image postprocessing tools is also essential, as signal and contrast differ from the adult brain. Appropriate methodologies then make it possible to explore multiple neurodevelopmental mechanisms in a precise way, and assess changes with age or differences between groups of subjects, particularly through large‐scale projects. Although MRI measurements only indirectly reflect the complex series of dynamic processes observed throughout development at the molecular and cellular levels, this technique can provide information on brain morphology, structural connectivity, microstructural properties of gray and white matter, and on the functional architecture. Finally, MRI measures related to clinical, behavioral, and electrophysiological markers have a key role to play from a diagnostic and prognostic perspective in the implementation of early interventions to avoid long‐term disabilities in children.

**Evidence Level:**

2

**Technical Efficacy Stage:**

1

How can the baby develop its amazing cognitive abilities and its intellectual functioning? Why do 5% to 10% of adults have neurodevelopmental disorders such as autism or dyslexia? Answering these questions requires a better understanding of how the human brain develops from the early stages. Studies of animal models are insufficient insofar as the development of the human species differs from that of other mammals by the short duration of pregnancy compared to the period between birth and adulthood. Although the newborn's brain has an early and relatively specialized organization, its immaturity allows postnatal modeling by the multiple experiences and learning that the infant is exposed to. The developing brain further shows a high degree of plasticity following early disruptions, particularly regarding networks that mature late in childhood.

Understanding the alterations to normal neural development that manifest as behavioral disorders in childhood is essential to address this important public health issue. Improving our knowledge of these mechanisms requires linking brain and behavioral changes *in vivo*, as *postmortem* studies are intrinsically limited by the inability to assess relationships with functional outcome, the scarcity of samples, the reasons for the decease of available specimens, and by tissue fixation. The use of noninvasive neuroimaging methods such as magnetic resonance imaging (MRI) has revolutionized our knowledge in this field over the past 20 years. The objective of this review article is to highlight recent MRI studies of the neonatal brain, focusing on the main methodological challenges and on the main neuroscientific insights. To our knowledge, no review article has so far addressed these issues jointly for the different MRI techniques available in newborns (anatomical MRI, diffusion MRI, other quantitative techniques, functional MRI [fMRI], etc.). We propose here to describe the two aspects of data acquisition and postprocessing, as well as the complementarity of MRI techniques in relation to the different facets of brain development (morphological changes, microstructural. and functional maturation, etc.).

## An Overview of Early Developmental Mechanisms

Before describing the MRI methodological aspects, it is important to remember that even longitudinal neuroimaging measurements only indirectly reflect the complex series of dynamic processes observed throughout development at the molecular, cellular, and macro‐anatomic levels. The human brain develops slowly, from embryonic conception to early adulthood, based on multiple events occurring within a highly constrained but constantly changing context.^1^, ^2^ During gestation, the brain shows a sequential development of transient laminar compartments, from the center to the periphery: the proliferative zones (ventricular and subventricular zones), the intermediate zone (future white matter [WM]), the subplate, the cortical plate (future cortex), and the marginal zone.

Neural proliferation and migration are predominant during the first trimester of pregnancy, while axon and dendrite growth occurs mainly during the second and third trimesters. Subsequently, prolonged maturation phenomena are observed, with synaptogenesis and pruning mechanisms, myelination, neurochemical maturation, etc. Not all of these mechanisms occur independently, but they most likely interact over extended periods. While very early phenomena occur endogenously, guided by our genetic heritage, many of them depend on exogenous mechanisms and fluctuate according to the baby's environment *in utero* and after birth.^3^

At the macroscopic level, brain growth is intense in the last trimester of pregnancy and the first two postnatal years, with a significant increase in gray matter (GM) and WM volumes.^4^, ^5^ The volume of cortex increases from ~10 to ~150 cm^3^ between 18 and 39 weeks of gestational age (w GA: equivalent to postmenstrual age [PMA] after birth)^6^, ^7^ and from 200 to 600 cm^3^ between 1 and 24 months of postnatal age.^8^ It is essentially based on an exponential increase in the cortical surface area: from ~150 cm^2^ at 27w PMA^7^ to ~700 cm^2^ and ~2000 cm^2^ at 1 and 24 postnatal months, respectively.^9^ This goes with an increasing complexity of the brain morphology and the formation of gyri, primary, secondary, and tertiary sulci from 20, 32, and 40w GA, respectively.^10^, ^11^ Although underlying mechanisms are still widely discussed,^12^, ^13^ the folding of the cortex allows the increase in its surface area while maintaining reasonable connection distances (and thus information transmission times) between brain regions.

These intense macroscopic changes in volume, surface area, and folding are probably the visible markers of changes in the microstructure of the cortical plate (the future cortex), which evolves in relation to various mechanisms during the preterm and postterm periods. After neuronal migration, the growth of connections between neurons is initially intense and exuberant, with synaptogenesis, growth, and complexification of the dendritic tree structure. Synaptic development is based on a phase of intense proliferation from 15w GA, then on a phase of selective elimination (pruning) of redundant or poorly used connections, which allows only those connections that are functionally relevant to be maintained.^14^ In parallel, intracortical fibers become myelinated, mainly during the early postterm period.^15^ These mechanisms of connections growth, synaptic development, and myelination occur over different time periods, depending on the cortical regions,^1^ with the primary, unimodal, and multimodal associative regions showing different trajectories of maturation.

In interactions with the development of cortical regions, structural connectivity develops within the WM, through intense and intertwined processes of growth and maturation during pregnancy and early childhood.^1^, ^5^, ^16^ During the early preterm period (26–30w GA), projection and callosal fibers that had established connections with subplate neurons begin to invade the cortical plate and develop connections with neurons of the future layer IV. Limbic connections are well developed in the cingulate, entorhinal, and hippocampal regions, as well as some associative bundles (eg, inferior longitudinal fasciculus). During the late preterm period (31–36w GA), long and commissural associative bundles develop rapidly. At the time of full‐term birth, the main long‐distance fibers are in place, while short‐distance fibers (eg, U fibers) develop mainly in the first year after birth.^1^ These anatomical connections are further refined, with some reorganization, through different complementary mechanisms. After exuberant growth, unnecessary or redundant connections are removed during childhood through the pruning process, as observed for callosal fibers in nonhuman primates.^17^

In addition, the process of myelination stabilizes those connections that are functionally relevant and increases the speed of information transfer between distant brain regions. In the human brain, it occurs from the second part of pregnancy to the end of adolescence, with a peak during the first postnatal year.^15^, ^16^, ^18^, ^19^ The ages and rates of occurrence depend on the regions and networks, and the general pattern of myelination relies on a caudorostral gradient, a progression from the brain center to the periphery, in sensory and motor pathways before associative pathways. Whereas the number of neurons and microglia cells remains approximately stable in the postnatal period, the number of oligodendrocytes and astrocytes increases considerably in the WM during the first 3 years, reaching two‐thirds of the values measured in adults.^20^ The inhibitory role of oligodendrocytes and myelin on neurite growth may partly explain the lower plasticity of the adult brain compared with the developing brain.^21^ Again, maturation takes place over different periods and at different rates between functional networks that are organized early on,^22^ with asynchronous progression of myelination between brain regions.^16^, ^19^, ^23^

A wide range of neurodevelopmental disorders originate from early disturbances in these complex and varied mechanisms during the pre‐ and perinatal period. In this review article, in parallel with neuroscientific studies targeting the understanding of typical development, we aim to highlight the potential of MRI for the diagnosis and prognosis of disorders related to prematurity and common neonatal insults.

## Common Methodological Challenges in Neonatal MRI

**FIGURE 1:**
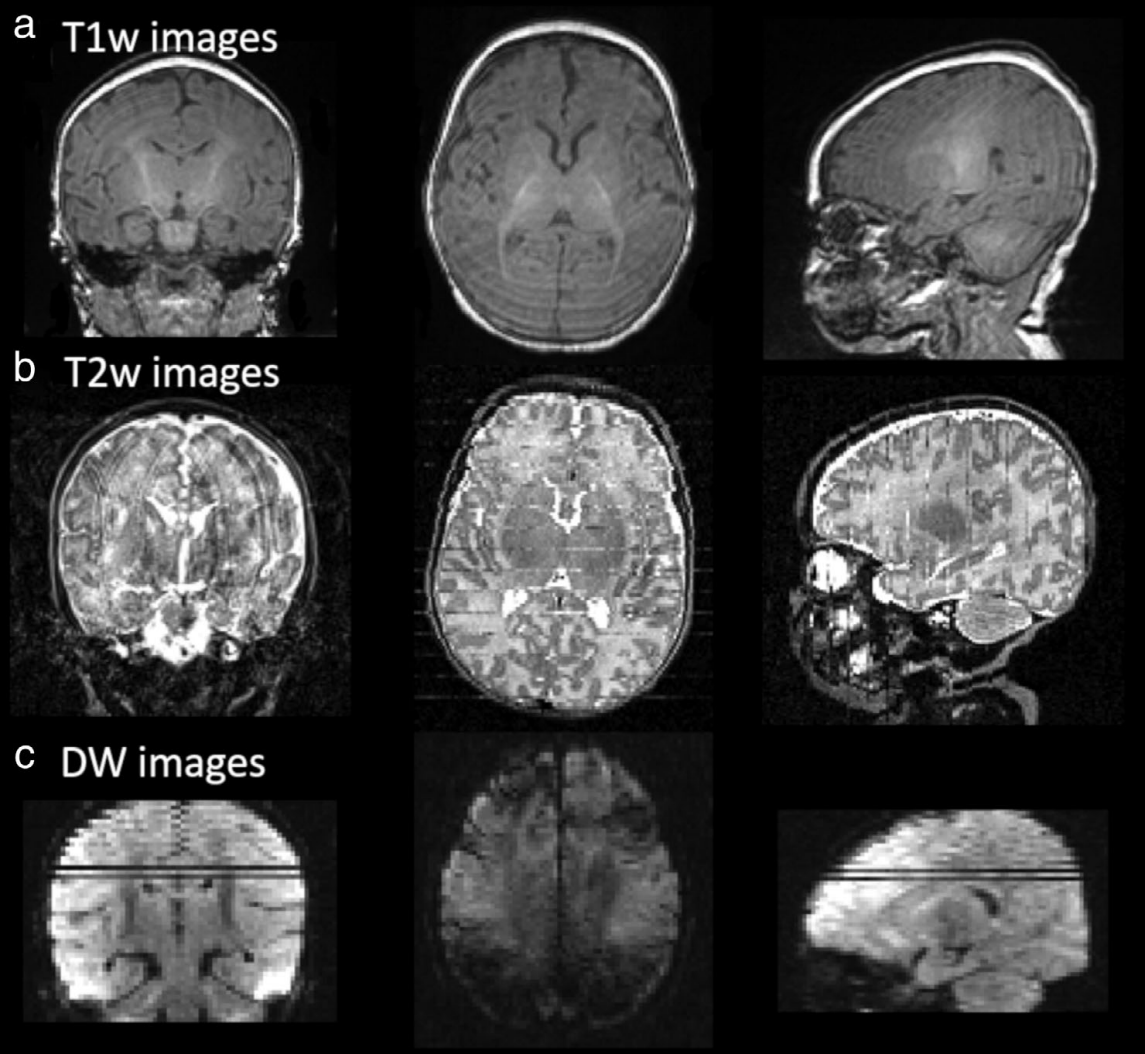
Typical motion artifacts on newborn images. Artifacts are presented in the different slice orientations, for T_1_w images acquired in 3D **(a)**, T_2_w images acquired in coronal plane **(b),** and DW images acquired in axial plane **(c)**.

Most MRI studies in neonates and infants now use 1.5 or 3T MRI scanners. Despite more significant associated radiofrequency radiation, 3T neuroimaging does not induce any significant increase in temperature.^24^ Nevertheless, this population of subjects poses multiple constraints that make research on the developing brain more complicated than in adults and also more challenging. In most centers, healthy full‐term infants cannot be sedated for research purposes, but only for clinical indications. This is problematic, since images are very susceptible to motion (Fig. 1). Different centers have adopted different approaches to address motion, with some groups choosing to use short acquisition sequences that can be run during a protocol not exceeding 30–45 minutes, and others using motion‐tolerant acquisition and reconstruction approaches explicitly designed for this hard‐to‐image population.^25^, ^26^, ^27^ The use of special settings (eg, hearing protections such as dedicated earphones or earmuffs; limiting the slope of the magnetic field gradient rise) also reduces the scanner noise to some extent and provides additional ear protection.

The small size of the brain structures is also an issue, and increasing the image spatial resolution is required to avoid significant effects of partial voluming. Besides, incomplete maturation of the infant's brain leads to different tissue characteristics than those of the adult brain, resulting in different values of MRI characteristics (eg, relaxation times: longitudinal relaxation time [T_1_] and transverse relaxation times [T_2_], diffusivities measured in diffusion MRI). Signal inhomogeneities and variability in these characteristics are observed across brain regions, given the maturation asynchrony that we described above. This issue requires adapting the acquisition sequences to obtain relevant and sufficient image contrast. Using dedicated coils, with optimized size in relation to the size of the head to be imaged, also makes it possible to maximize the signal‐to‐noise ratio (SNR) in the images^28^, ^29^, ^30^ with a gain greater than x2, depending on the brain regions and their proximity to the coil elements. Considering dedicated image postprocessing tools is also required to deal with the signal and contrast specificities of newborn images. Imaging studies of the developing brain therefore rely on specially trained teams with dedicated expertise in both acquisition and data processing.^31^ The last 20 years have seen an increasing number of clinical and research teams interested in this subject, as well as the emergence of large‐scale projects (eg, the “developing Human Connectome Project” [dHCP] targeting fetuses and newborns between 20 and 44w PMA; the “Utrecht Baby MRI Youth Project” targeting fetuses and newborns between 30 and 44w PMA; the “Baby Connectome Project” [BCP] targeting children between birth and 5 years of age). This has allowed for the intensive development of specialized methodologies, for instance, to address infant motion.^32^, ^33^

Given the increasing number of publications on this topic, this review article could not be exhaustive but aimed to report representative recent research and clinical studies conducted using different techniques in neonates and infants. We selected the most recent publications, focusing mainly on those of the last 10 years, published before September 2019, which we felt illustrated major advances in this field. Wherever possible, we have privileged the citation of recent review articles and the reader is invited to turn to these to delve more deeply into a particular issue. We have tried to cite articles from all groups that have provided relevant work on this topic in recent years, and to limit the number of citations from the same groups when the corresponding articles covered the same topic. This review article is organized by technique as follows: Anatomical and Relaxometry MRI, Diffusion MRI, Other Quantitative and Multiparametric Methods, and Functional MRI.

## Anatomical and Relaxometry MRI

### Developmental Specificity and Methodological Challenges

**FIGURE 2:**
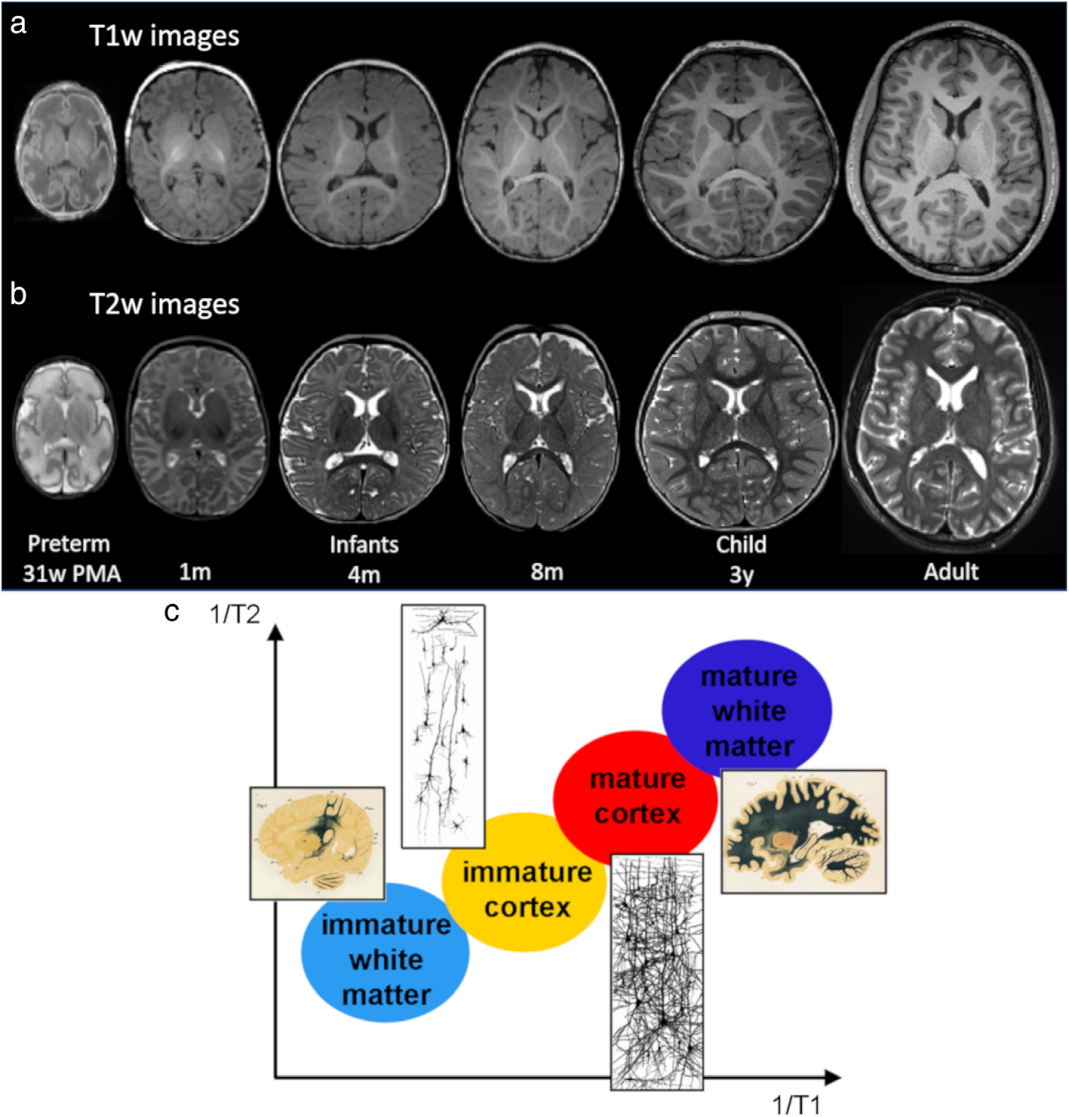
Evolution of anatomical images during development. T_1_w **(a)** and T_2_w **(b)** images are presented for a preterm newborn at 31 weeks of postmenstrual age, term‐born infants at 1, 4, and 8 months of age, a 3‐year‐old child (with a small occipital dysplasia lesion), and a young adult. Developmental changes in T_1_w and T_2_w contrasts are explained by age‐related decreases in T_1_ and T_2_ relaxation times that are more intense in the WM than in the GM because of the myelination process **(c)**. Adapted from Ref. ^16^.

Anatomical MRI weighted by T_1_ or T_2_ relaxation times must face the challenge of different water and fat content in the neonatal brain compared with adults, resulting in different signal intensities in newborns and infants. These contrasts evolve with brain maturation, and successive stages are generally described^16^: 1) the infantile pattern (0–6 months), showing a reversal of the normal adult contrasts (T_1_‐weighted [T_1_w]: lower WM intensity than GM intensity; T_2_w: higher WM intensity than GM intensity); 2) the isointense pattern (8–12 months), characterized by a poor contrast between GM and WM; 3) the early‐adult pattern (>12 months) (T_1_w: higher WM intensity than GM intensity; T_2_w: lower WM intensity than GM intensity) (Fig. 2a,b). Both T_1_ and T_2_ decrease with maturation processes and parallel the decrease in water content. The changes in T_1_w and T_2_w contrasts are due to the fact that T_1_ and T_2_ decrease more strongly in WM than in GM because of myelination processes (Fig. 2c), to the extent that water molecules located within the myelin sheath have the shortest T_1_ and T_2_ characteristics.^16^ The time courses of T_1_ and T_2_ decreases are different, and two distinct mechanisms might be distinguished in the WM: the change in water molecules compartmentalization (impacting mostly T_1_ shortening during the “premyelinating” state), and the increase of protein and lipid contents with the chemical maturation of the myelin sheath (leading mostly to T_2_ shortening). Thus, changes in contrasts are observed on T_1_w images before T_2_w images. In neonates and infants during the first 6–8 postnatal months, T_2_w images are generally preferred for the delineation and segmentation of GM and immature WM, whereas T_1_w images are used to identify the myelinated WM. Several studies have aimed to optimize MR sequence parameters to improve the image contrast between GM and WM. This concerns in particular the inversion times of 3D T_1_w sequences at 3T in neonates^34^ and infants.^35^

The spatial resolution of T_1_w and T_2_w images is also a major issue for the accurate delineation of brain structures. 2D or 3D sequences can be considered, but the contrast provided is not necessarily the same. For research purposes, some teams promote the acquisitions of high‐resolution 2D images in three complementary planes with overlapping slices (eg, axial, coronal, sagittal) in order to use a “super‐resolution” approach and reconstruct a more resolved volume.^36^ Others aim to acquire images with isotropic resolution (~1 mm) in the three directions of space.^37^

Beyond data acquisition, the processing of anatomical images differs between the brains of premature newborns, infants, and adults. As detailed below, several dedicated tools have been proposed in the recent years for the segmentation of tissues, the morphological evaluation of brain growth, and for computational neuroanatomy of infant brains.^38^ The combination of images with different contrasts (T_1_w and T_2_w) has been tested to better distinguish between tissues and between regions with different maturation, but so far the benefit does not seem so obvious.^39^ The potential of deep‐learning approaches has begun to be explored but the signal variability across the first postnatal year remains a major challenge.^40^

### Morphometric Studies

#### Measuring brain growth

To segment brain tissues, different methodologies have been proposed, which can be classified into unsupervised, parametric, classification, atlas fusion, and deformable models.^41^ In the recent context of the dHCP project, a fully automated pipeline has been proposed to process anatomical images of preterm and full‐term newborns from 28–45w PMA and provide reliable cortical surface extraction and inflation.^36^ Longitudinal imaging can further help to address the issue of changing contrast in full‐term infants from 2w to 18 months of postnatal age, by incorporating longitudinal constraints and providing temporally‐consistent and accurate surfaces.^38^ In preterm infants from 30–40w PMA, larger volume and surface changes have been observed in the occipital lobes than in the other lobes.^42^ During the first postnatal month, full‐term infants show different age‐related increases in GM and WM volumes,^39^, ^43^ which is to reflect the early intense growth in GM compared with the more protracted WM growth.

In parallel, the cortical thickness develops dynamically in a spatially heterogeneous way, but measuring it remains a challenge, given the image spatial resolution. A dedicated pipeline of longitudinal data and an advanced multivariate analysis method have characterized the temporal evolution of cortical thickness from 1–24 months of postnatal age and highlighted a developmental regionalization into structurally and functionally meaningful regions.^44^ Each shows a specific increasing–decreasing pattern, with thickness values ranging between 2 and 3.5 mm, and peaks with maximal thickness at varied ages in the second year. By 2 years of age, thickness is on average 97% of adult values, whereas surface has only reached two‐thirds.^9^ It seems that slightly thicker cortices at 1 and 2 years of age (within a normal range of values) might confer some cognitive advantage in infancy and toddlerhood.^45^

Nevertheless, the interpretation of changes in cortical thickness as measured by MRI is subject to discussion, as they are based on images that also change in contrast with maturation, particularly during the first 2 postnatal years.^5^, ^16^ It was first suggested that the age‐related changes in the apparent MRI thickness (with an initial increase followed by a decrease with age) could be related to synaptic overproduction and pruning. However, synapses only represent a small fraction of the total cortical volume, and modifications at the level of dendrites, cell bodies, and fibers might be the major factors influencing the measured thickness.^1^ Combining the thickness measures with microstructural markers of cortical maturation (see subsections Microstructural Studies and Microstructural Measures in the Gray Matter, below) might help to better understand the underlying processes. Furthermore, since movement artifacts might vary with age and bias the measured apparent thickness, data quality and quality control procedures have a significant impact on the identified age‐related trajectories.^46^ Overall, recent studies in older children have suggested that MRI apparent thickness decreases in most cortical areas by the age of 3 years (see Ref. ^47^for a detailed review).

#### Mapping the brain folding process

**FIGURE 3:**
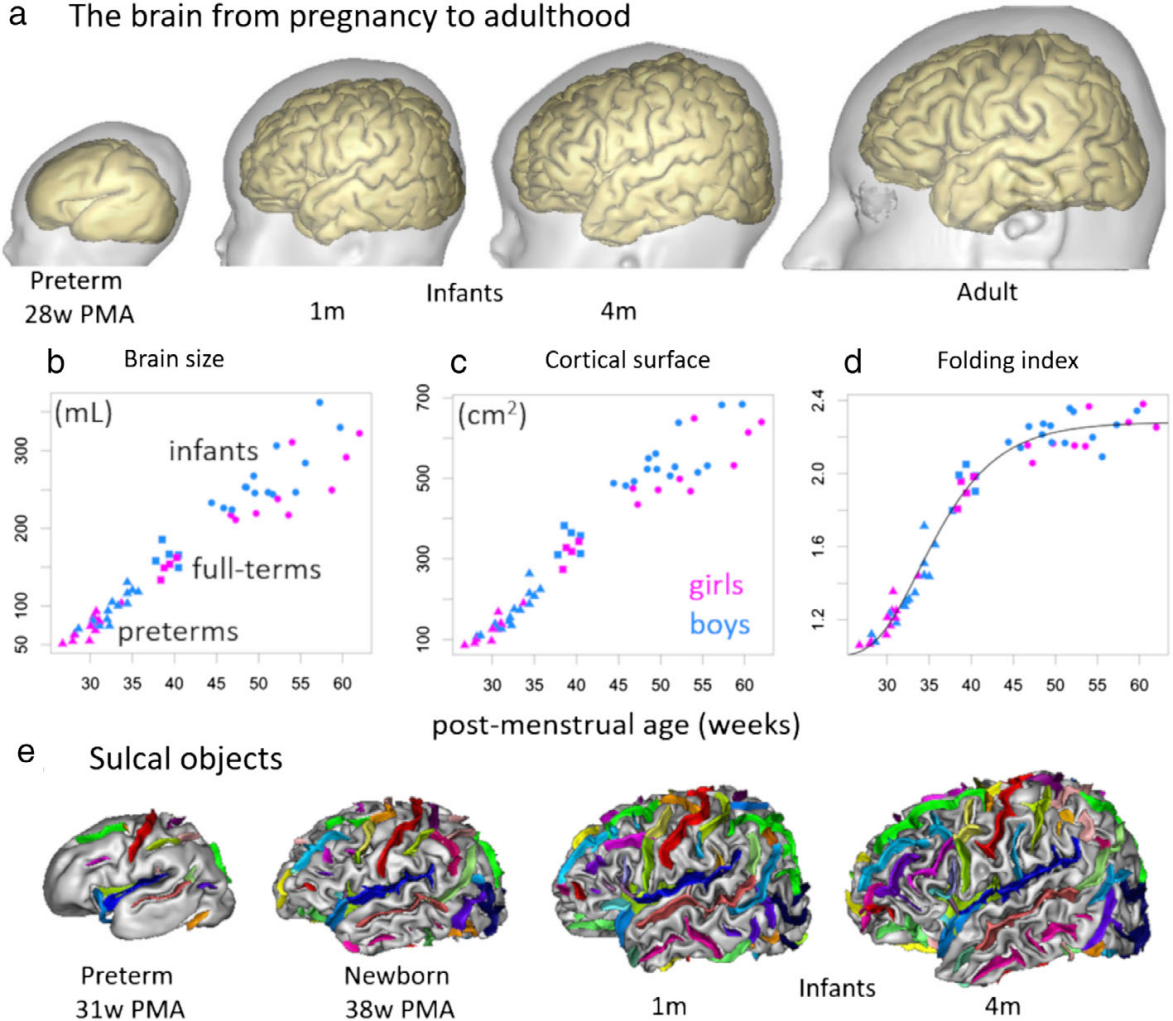
Brain morphological changes from pregnancy to adulthood. Cortical surface reconstructions are presented for a preterm newborn at 28 weeks of postmenstrual age, term‐born infants at 1 and 4 months of age, and a young adult **(a)**. Around birth, cerebral development is characterized by important increases in brain size **(b)**, cortical surface area **(c),** and folding index (**d**: ratio between inner cortical surface area and the area of the closed surface) as measured in preterm and full‐term newborns and infants (adapted from Ref. 37). Based on cortical and brain surfaces, it is possible to identify “sulcal objects” (**e**: illustrations for a preterm newborn at 31w PMA, a full‐term newborn at 38w PMA, and infants at 1 and 4 months of age) which can be used to perform morphological measurements during development^48^ or to register brains of different sizes.^49^

Based on the 3D reconstruction of cortical surfaces (Fig. 3a), it is possible to compute a wide variety of folding measures (eg, depth, gyrification index, measures based on curvature).^37^, ^50^, ^51^ These measures evolve with the infants' age, through complex relationships with brain size and cortical surface area (Fig. 3b,c). In infants from 27–62w PMA (~5 months of postterm age), a nonlinear increase in the gyrification index is observed, with major changes during the preterm period (before 40w PMA) and a slowdown afterwards^37^ (Fig. 3d). Nevertheless, the gyrification index still increases afterwards during the first 2 years, but to a lesser extent, with high‐growth regions in association cortices and low‐growth regions in sensorimotor, auditory, and visual cortices.^38^

Beyond the temporal aspect, studies support the spatial heterogeneity in folding, with different rates across regions. The spatial‐frequency structure of cortical patterns was further detailed with a spectral analysis of gyrification, and three successive waves were identified over the 27–62w PMA period, which might correspond to primary, secondary, and tertiary folding.^37^ This approach could make it possible to distinguish between the folds that formed successively, at the individual scale. Furthermore, maps of cortical expansion have highlighted regional differences in growth that are consistent with the emergence of new folds during the 28–38w PMA period.^52^ Between 30w PMA and term‐equivalent age (TEA), the major primary sulci show an intense growth (Fig. 3e), and sulci developing the earliest seem to be the most affected by clinical factors such as birth weight, multiple pregnancy, or prolonged mechanical ventilation.^48^

As in adult studies, the methods used in newborns to quantify brain growth with volumetry and morphometry do not necessarily yield convergent results, making comparisons between studies difficult. No method has been fully validated so far because there is no ground truth and because MR images acquired in the neonate are inherently limited in terms of tissue contrast and spatial resolution. Therefore, biological interpretation of the results must be performed with caution.

#### REGISTERING BRAINS AT DIFFERENT DEVELOPMENTAL STAGES

In studies based on voxel‐based statistics (eg, for morphometry, fMRI), group analyses or group comparisons require aligning the brains of the subjects in a common space by registering and spatially normalizing them. This is particularly challenging for brains with different sizes and folding patterns. In recent years, a few approaches have been proposed. Some use the segmented tissue maps instead of raw T_1_w and T_2_w images, and consider both these maps and cortical surfaces in the nonlinear registration process.^53^ Similarly, a two‐step landmark‐based strategy allowed to registering the brains of preterm newborns, infants, and various databases of adults.^49^ The DISCO method (diffeomorphic sulcal‐based cortical registration) can be used to embed sulcal constraints in a registration framework used to initialize the DARTEL step (diffeomorphic anatomical registration using exponentiated Lie algebra; implemented in SPM software [MatLab, MathWorks, Natick, MA]) (Fig. 6). Using anatomically constrained multimodal surface matching (MSM) also appeared reliable to provide accurate correspondence between longitudinal cortical reconstructions of the same infants.^52^ These methodologies can be used to set up age‐dependent templates and spatiotemporal atlases, as shown in 36‐44w PMA newborns with MSM registration driven by the cortical folding.^55^ Characterizing the anatomical variations in time‐dependent changes and the disease‐related alterations requires complementary high‐resolution atlases dedicated to the developing brain.^56^

#### PREDICTING THE INFANTS' development based on early brain morphometry

Several studies of early brain growth and folding (during the equivalent of the third trimester of pregnancy) have been based on premature newborns, who cannot be considered a “normal” developmental model. Folding deviations have been observed in different groups of preterm infants (eg, with/without brain injury, extremely/moderately preterm) compared with full‐term infants.^37^, ^50^, ^57^ Furthermore, even if the results are not fully consistent, differences in brain growth trajectories and morphology have been highlighted in fetuses and preterm infants without brain lesions.^51^, ^58^ Studies in premature newborns are nevertheless essential from a clinical perspective, since they could play a diagnostic or even prognostic role in babies at risk of developing a sensorimotor or cognitive disorder. For instance, longitudinal brain morphometry in the early preterm period (at birth or at 30w PMA) and at TEA has to some extent allowed identifying infants at risk of cognitive and motor impairments based on machine‐learning approaches,^59^, ^60^ although socioeconomic status remains the most powerful predictor of outcome.

### Microstructural Studies

In parallel with morphometric and morphologic changes, brain tissues show major changes in microstructural characteristics. Together with the development of dendritic arborization, fiber myelination, changes in water content, and other processes, T_1_ and T_2_ change intensively in GM and WM.^16^, ^61^ Nevertheless, to understand maturation processes or disturbances, T_1_w and T_2_w signals cannot be directly compared across regions or across individuals because of the variability between examinations related to technical parameters, head size, and position inside the coil. To provide such comparisons, either signals may be “normalized” for each subject, or T_1_ and T_2_ relaxation times may be quantitatively measured.

It was first proposed to relate the T_2_w signal in each voxel to the one of local cerebrospinal fluid (CSF), which is not supposed to vary between regions and throughout development.^62^ In infants younger than 4 months of age, some maturation asynchrony was observed between primary and associative cortical regions of the language network. More recently, in the continuity of studies conducted in adults, the T_1_w/T_2_w ratio has been proposed as a marker of myelination. In neonates, this improves the contrast of early myelinating WM structures (eg, posterior limb of the internal capsule, corticospinal tract, optic radiations).^63^ Between 36 and 44w PMA, the T_1_w/T_2_w ratio also increases in cortical regions, suggesting intense maturation and differences between sensorimotor and associative regions.^55^

**FIGURE 4:**
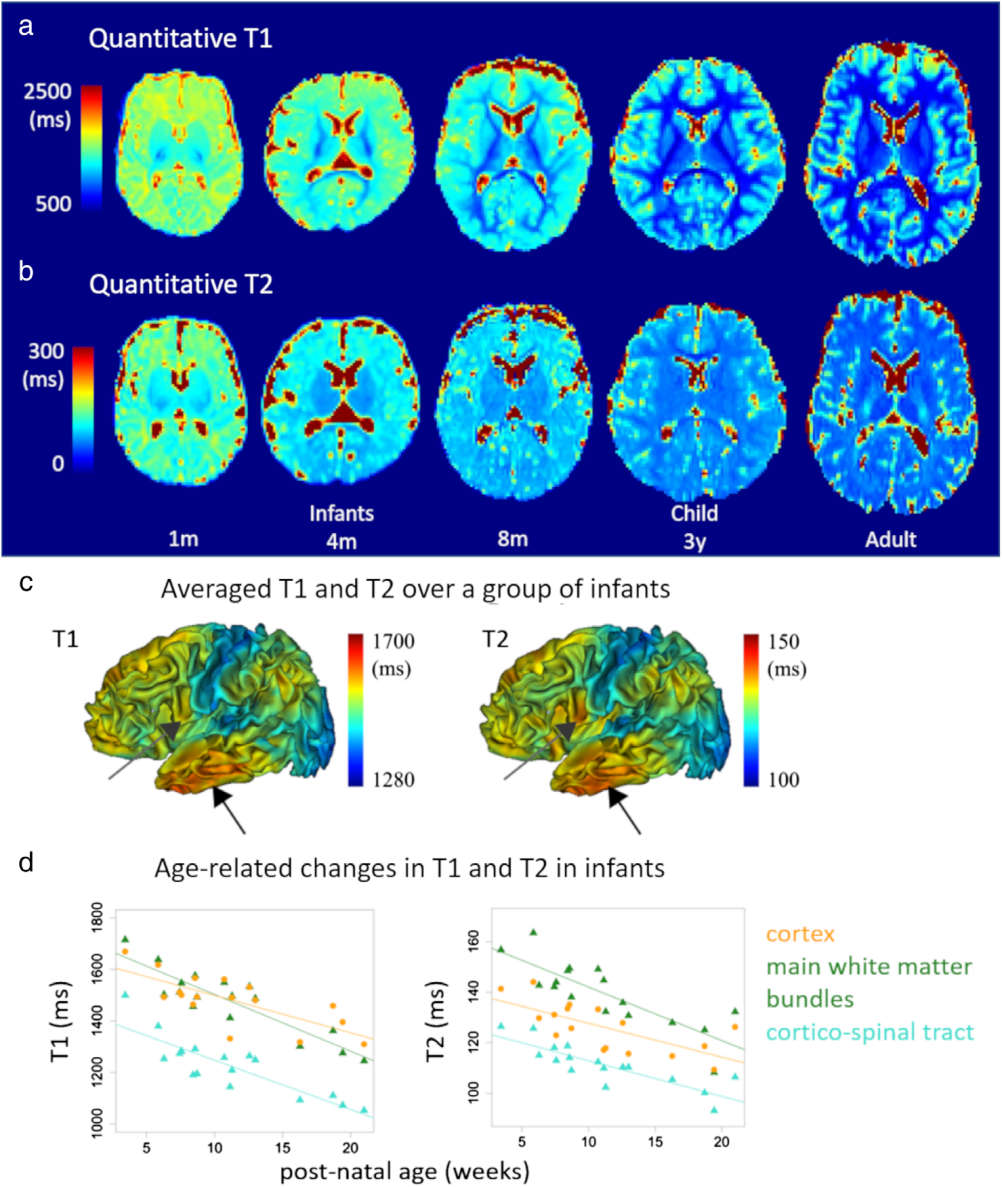
Evolution of quantitative T_1_ and T_2_ maps during development. T_1_ **(a)** and T_2_ **(b)** maps are presented for term‐born infants at 1, 4, and 8 months of age, a 3‐year‐ old child, and a young adult (same subjects as in Fig. 2). Differences in T_1_ and T_2_ times are observed across cortical regions during infancy (average over a group between 1 and 5 months of age), suggesting early microstructural differences across cortices **(c)**. T_1_ and T_2_ decrease with the infants' age, at higher rates in the WM than in the cortex **(d)**. Adapted from Refs. ^16^, ^68^.

Quantitative measures of T_1_ and T_2_ relaxation times are a more principled approach to compare between brain regions or between infants. However, this requires the acquisition of additional sequences, making the protocol longer and thus challenging in nonsedated infants. Different methods have been proposed in recent years to reduce the acquisition time. Using 3D MP2RAGE (magnetization prepared 2 rapid acquisition gradient echoes) sequence^64^ or 3D SPGR (spoiled gradient recalled) sequence with various flip angles^65^ provides reliable quantification of T_1_ times. In preterm newborns imaged from birth to TEA, T_1_ measures show a gradual decrease with age in different regions of WM and GM, including the cortex and central gray nuclei.^64^, ^65^ Turbo/fast spin‐echo sequences with multiple echoes allow quantitative T_2_ times to be determined, which decrease continuously with increasing age, especially during the first postnatal year (Fig. 2d).^66^ With echo‐planar imaging (EPI) spin‐echo sequences with different sequence parameters (several inversion times [TI] or echo times [TE]), T_1_ and T_2_ values could be computed over the whole brain of infants and with good spatial resolution^67^ (Fig. 4a,b). But the presence of EPI‐related geometric distorsions required correcting the resulting maps in a nonlinear way to match anatomical images^69^ and provide reliable measures in the cortical mantle^68^ (Fig. 4c). The BCP project used an MR fingerprinting approach to provide simultaneous quantification of T_1_ and T_2_.^70^ Marked increases in R1 (~1/T_1_) and R2 (~1/T_2_) were observed in several WM regions from birth to ~20 months of age, followed by slower increases thereafter.

When interpreting T_1_ and T_2_ times in terms of maturation, it is important to consider their variability between regions in the mature state (different brain regions might show variable values also in the adult brain). Simply comparing values between immature regions, therefore, does not allow drawing conclusions relating to advanced or delayed maturation of one region compared with another.^16^ To do this, it is necessary to have a reference per region, measured, for example, on an adult cohort with a comparable MRI protocol,^67^ or to measure their maturation trajectories according to age.^68^

## Diffusion MRI

Diffusion MRI is another major technique to study the developing brain of newborns and infants. As for previous structural approaches, it requires optimizing the acquisition parameters, and it is exquisitely sensitive to subject motion.

### Developmental Specificity and Methodological Challenges

Because of the high water content and the low myelination of the brain, the diffusion properties are very different (ie, higher diffusivity values and lower anisotropy values) in the immature brain compared with the brains of children and adults.^16^ EPI sequences are generally used for data acquisition, and the recent advent of a multiband acceleration technique is an opportunity to further reduce the acquisition time. Whereas whole‐brain diffusion tensor imaging (DTI) can be performed in a reasonable time (<5 minutes), high angular resolution diffusion imaging (HARDI) and multicompartmental diffusion techniques (as detailed below) require the long acquisition of multiple shells (data for several b‐values) with multiple diffusion gradient directions to provide an accurate estimation of the diffusion model and improve SNR. For the dHCP project, a dedicated time‐efficient acquisition framework (total protocol <20 minutes has been proposed).^71^ The protocol for the BCP project also aims to take into account the cohort age range (0–5 years) by optimizing imaging parameters and considering different imaging procedures for asleep and awake subjects.^72^ Furthermore, it is possible to generate specific schemes of diffusion gradient directions according to movement scenarios expected during the data acquisition.^73^

In addition, preprocessing is a major step in studies of neonates. Before analyzing diffusion data in a reliable way, correcting for motion artifacts is a prerequisite, whether it concerns intraslice or intravolume artifacts resulting from a sudden movement, or spatial drifts observed between volumes corresponding to different gradient directions.^74^ For the dHCP project, an automatic processing pipeline has been validated to deal with neonatal‐specific issues in this large dataset.^75^

**FIGURE 5:**
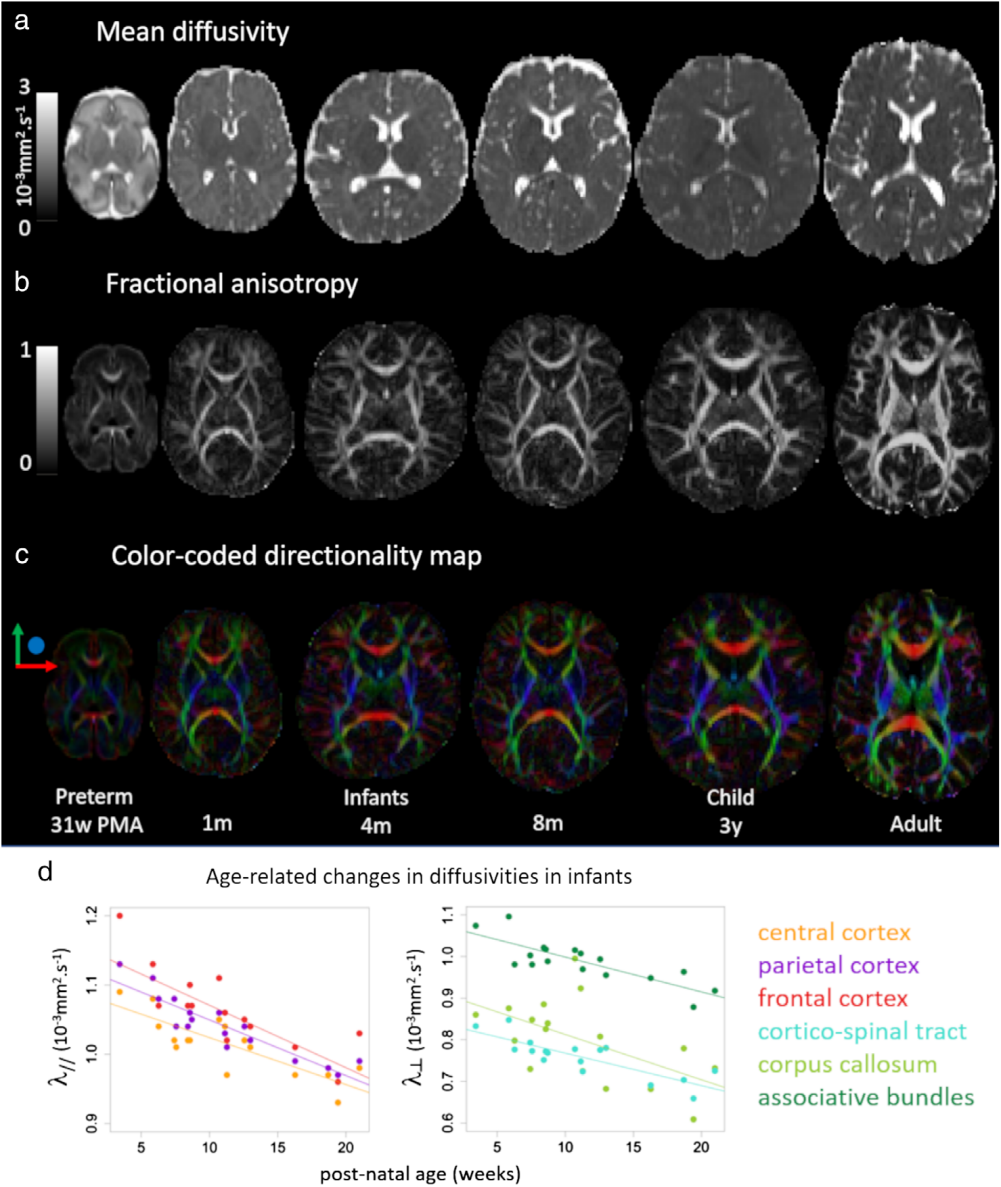
The evolution of DTI maps during development. Mean diffusivity **(a)**, fractional anisotropy **(b),** and color‐coded directionality **(c)** maps are presented for a preterm newborn at 31 weeks of postmenstrual age, term‐born infants at 1, 4, and 8 months of age, a 3‐year‐old child, and a young adult (same subjects as in Fig. 2). Axial (λ_//_) and radial (λ_┴_) diffusivities are markers sensitive to the maturation of GM and WM, respectively, which decrease with the infants' age in different ways across regions and tracts **(d)**. Adapted from Ref. ^16^.

**FIGURE 6:**
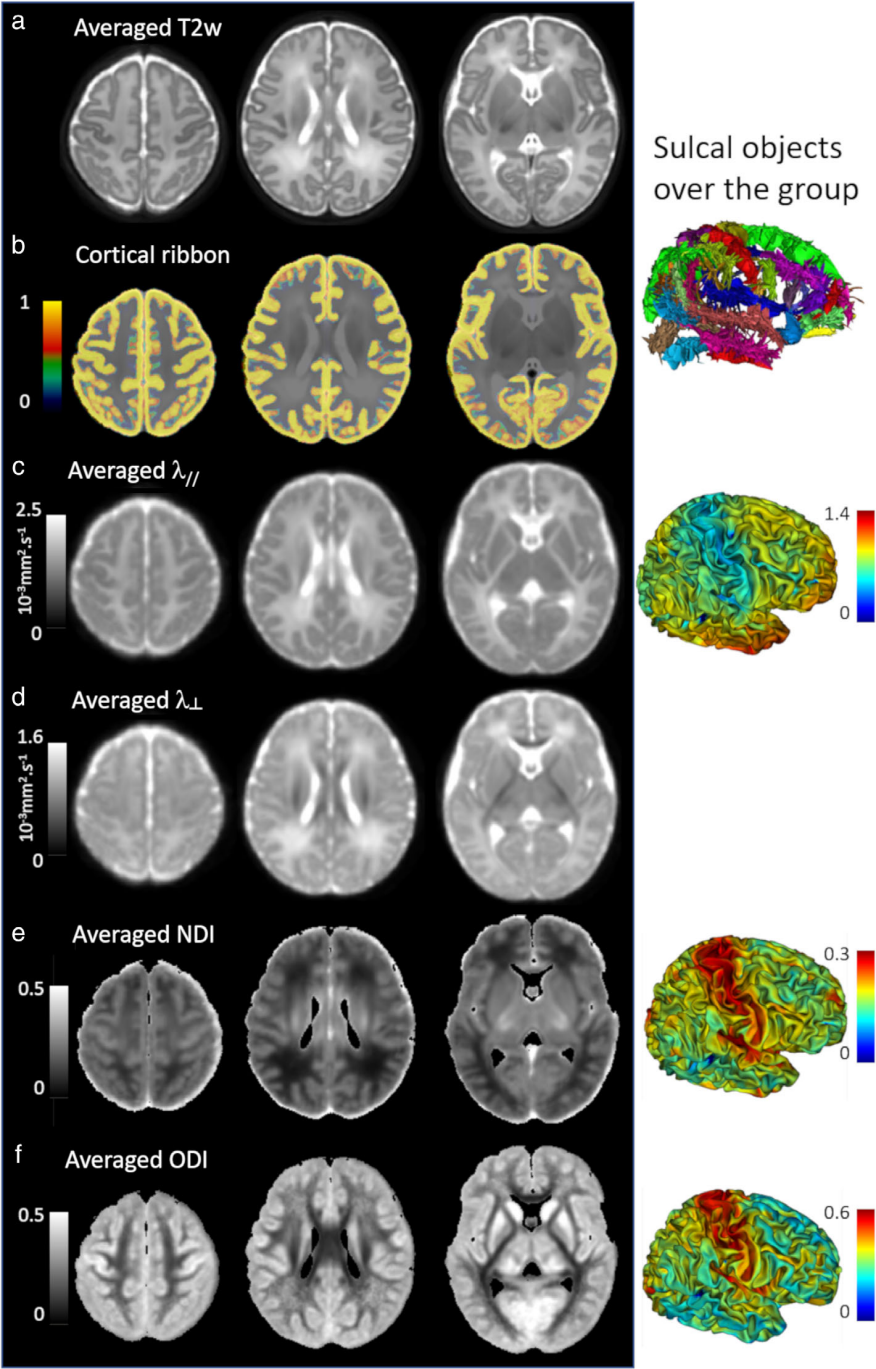
Averaged maps over a newborns' group. The DISCO+DARTEL registration framework^49^ was used to register data of 40 full‐term newborns (first dHCP release: PMA between 37 and 44 weeks). The averages of registered images and maps are presented for T_2_w images **(a)**, cortical ribbons **(b)**, DTI maps (**c,d**: axial λ_//_ and radial λ_┴_ diffusivities), and NODDI maps (**e**: neurite density index NDI, **f**: orientation dispersion index ODI). The right column shows the registered cortical sulci over the group as well as the averaged indices on the cortical surface, suggesting microstructural differences across cortical regions. Adapted from Ref. ^54^.

Modeling the diffusion signal is also a problem in itself. Given the required acquisition time, DTI is still the most commonly used approach to analyze diffusion data of newborns^61^, ^76^ (Fig. 5a,b). Nevertheless, the associated limitations are numerous and many pitfalls have been identified at the different stages of data postprocessing (see, for instance, Ref. ^77^for a detailed review in adults), which imposes caution in the interpretation of the observations obtained with this method. Thus, more elaborate models have been proposed recently to analyze multishell HARDI data,^61^ such as neurite orientation dispersion and density imaging (NODDI)^78^, ^79^, ^80^ (Fig. 6e,f) or diffusional kurtosis imaging (DKI).^81^ Decomposing the signals into multiple components from multiple tissues with spherical deconvolution is a promising approach to provide reliable longitudinal investigations of WM maturation.^82^

Finally, as illustrated for preterm newborns at 33, 36, and 39w PMA,^83^ defining age‐specific diffusion templates and atlases is required to account for the dramatic morphological changes throughout development and perform group comparisons or detect anomalies in a reliable way.

### Microstructural Measures in GM

Diffusion MRI allows very precise *in vivo* exploration of the GM microstructure. During the preterm period, the laminar organization of the cerebrum can be delineated, since compartments such as the cortical plate, subplate, and central gray nuclei show different diffusion properties of water molecules.^65^, ^84^, ^85^ At the cortical plate level, these properties evolve in a complex way with age. Using DTI, different groups have observed an early anisotropy and a radial orientation of the main tensor eigenvector from 27w PMA, possibly relying on the early presence of radial glia fibers and apical dendrites of pyramidal neurons.^61^, ^86^ This transient microstructure has recently been quantified by a “radiality index” measuring the local directional coherence between the diffusion tensor and the cortical surface.^87^ Subsequently, diffusion within the cortical plate becomes isotropic (ie, diffusion anisotropy decreases) with elongation and complex branches of neural connections (eg, basal dendrites of pyramidal neurons, thalamocortical fibers). This decrease in DTI anisotropy in the cortex appears to stabilize around term‐equivalent age, whereas diffusivity indices continue to decrease^78^ (Fig. 5d). Several competitive microstructural mechanisms could explain the observed age‐related changes, such as decreased neuronal density associated with programmed cell death, increased glial and organelle cells, complexification of the neuropile between cellular bodies, decreased water content, etc., rather than overproduction and pruning of synapses that only represent a small volume within the GM.^1^ The NODDI model might be used to better understand the progression of these mechanisms during development,^78^, ^88^ as the neurite density index informs about the cellular and organelle density, and the orientation dispersion index on geometrical microstructure. DKI might also provide valuable insights on the cortical microstructure, with continuous decrease in mean kurtosis over the preterm period.^89^ More studies are needed to systematically compare the markers provided by the different diffusion models in the developing brain according to the age of newborns and the cortical regions.

Studies using these more or less complex models converge to show differences in microstructural changes between cortical regions over the preterm period,^90^, ^91^ with an apparently less complex microstructure (ie, higher DTI anisotropy and diffusivity values) but more intense age‐related changes in gyri than in sulci, and in frontal lobes than occipital.^61^, ^86^ Furthermore, the occipital lobe showed the fastest changes in the radiality index^87^, ^90^ and mean kurtosis.^89^ After 38w PMA, the increase in NODDI neurite density index is still observed but in restricted regions (primary motor and sensory regions).^78^

Using both DTI and NODDI models, heterogeneities in cortical microstructure have also been observed between cortical regions of the whole brain in full‐term newborns (Fig. 6). And some differences have been described within a functional system; for instance, in auditory and linguistic regions of preterm newborns between 26 and 42w PMA^92^ and infants between 1 and 5 months of age.^93^ Comparing the microstructure of similar cortical regions across the left and right hemispheres, on a voxelwise basis, was also made possible in infants through the careful registration of native and flipped brains and DTI maps, with a two‐step matching strategy of sulci and cortical ribbons to compensate for morphological asymmetries.^94^ This framework highlighted asymmetrical microstructural organization in specific sensorimotor and language regions.

Although the relationships are not well established yet, the complex changes in cortical microstructure as assessed with DTI and NODDI might be related to the gyrification process^78^ and to the maturation of associated WM tracts.^89^ These patterns seem to differ between preterm infants at TEA and full‐term newborns.^92^, ^95^ DTI also allows the microstructural exploration of central GM nuclei in the developing brain,^61^, ^65^, ^96^ but there are still few analyses provided with more comprehensive models such as NODDI or DKI.

### Exploration of WM Development

In addition to the study of GM microstructure, diffusion MRI is the technique of choice for mapping WM development (connectivity and maturation) in newborns and infants.^4^, ^16^

#### White matter connectivity

As early as the preterm period,^97^ the organization of main bundles is clearly delineated on DTI directionality maps showing the main direction of the diffusion tensor (Fig. 5c). In recent years, HARDI models and estimation of fiber orientation distribution functions (fODF) have allowed the precise visualization of crossing fibers such as in the corona radiata.^98^ A wide variety of tractography tools can be used to reconstruct the apparent trajectory of WM bundles in 3D. However, the ones based on simple diffusion models (eg, DTI that only considers a single fiber population per voxel) present major limitations, leading to biases such as false negatives (premature termination of a tract) or false positives (switch of a tract to a neighboring one).^99^ It is only by using the most sophisticated tools, requiring HARDI data and fODF estimation, that one can hope to explore structural connectivity with the best anatomical reliability. But this requires long acquisition times that are sometimes difficult to achieve in newborns.

**FIGURE 7:**
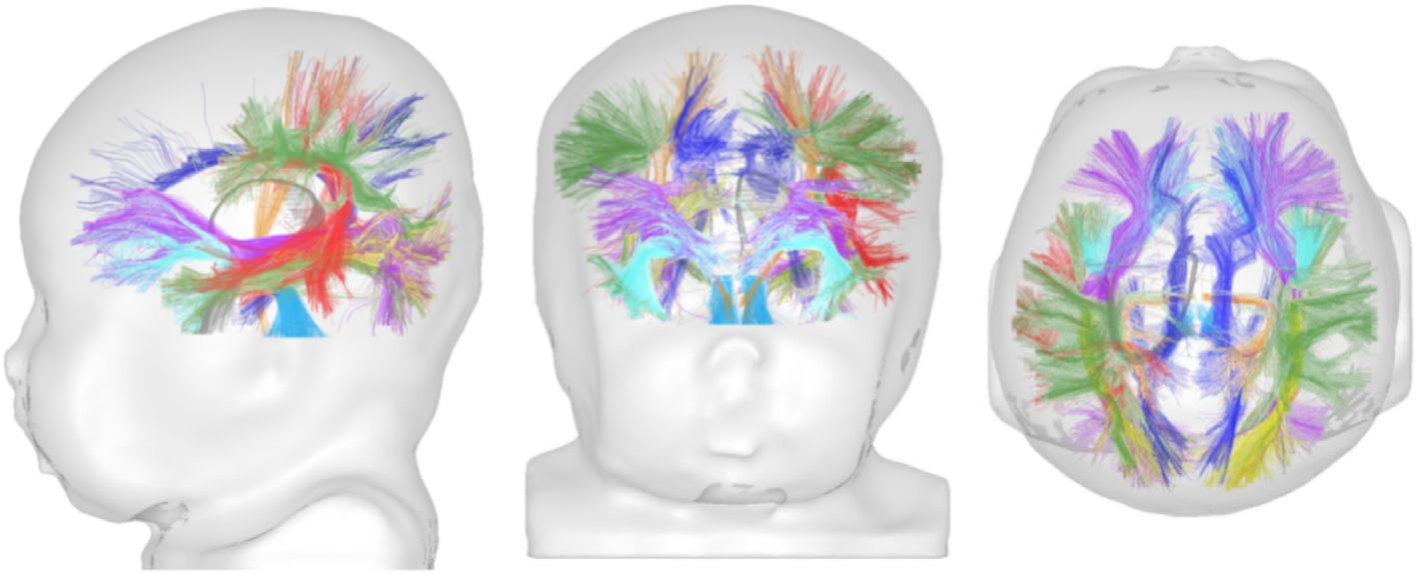
Tractography of the infant's WM bundles. Based on fODF estimation and tractography methods, the trajectory of the main WM bundles (projections, callosal tracts, limbic, and associative bundles) can be reconstructed, providing “virtual dissections,” as illustrated here in a 1‐month‐old infant.

In newborns and infants, current techniques have enabled reconstructing the main bundles despite their immaturity^67^, ^100^, ^101^: the commissural fibers of the corpus callosum, limbic bundles (fornix and cingulum), projection bundles (eg, cortico‐spinal and spino‐thalamic tracts, optical radiations, anterior arm of the internal capsule), and associative bundles (eg, external capsule, uncinate fasciculus, arcuate, inferior and superior longitudinal fascicles) (Fig. 7). During infancy and toddlerhood, the bundles morphology remains stable and a population‐specific atlas can be used for their identification with diffusion MRI.^102^ Automatic tractography tools, including, for instance, prior information about the anatomical neighborhood of pathways, have also been specifically designed for newborns.^101^, ^103^ Nevertheless, the biological interpretation of tractography reconstructions remains an issue, since not only axonal fibers participate, but also radial glia fibers and blood vessels, particularly during the preterm period.^104^ In addition, tractography methods based on diffusion MRI do not allow the measure of axonal pruning,^98^ which extends into the first few months after birth.^1^ This phenomenon could indeed lead to signal changes, but these may not be evident, as other changes in the opposite direction, mainly related to myelination, may dominate. In the future it would be interesting to take advantage of the differential maturation of bundles during infancy to evaluate their trajectory with greater reliability and specificity.^98^

In recent years the developing architecture of structural networks has also been detailed by whole‐brain connectome approaches^105^, ^106^ that rely on connectivity matrices measuring the degree of connections between pairs of brain regions. From 30w PMA, the structural connectome presents a “small world” modular organization, as in the adult brain,^107^ and some cortical nodes (the “hubs”) are intensely connected and form a “rich club” architecture.^108^ This topology is further refined with age,^107^, ^109^, ^110^ with an increase in overall efficiency and integration, a decrease in segregation, and a remarkable hierarchical order from primary to higher‐order regions.^105^ Nevertheless, these connectome methods and the tractography reconstruction techniques they use have potential biases that alter the biological accuracy of observations, as detailed in the adult brain.^111^ In particular, studies based on DTI probably describe sparse networks compared to actual ones described in animals, which could prevent the accurate delineation of rich‐club architecture in newborns.^112^

#### White matter maturation

As the connections develop, the WM fibers gradually become mature and functional through the process of myelination, whose successive steps can be measured with diffusion MRI.^16^, ^61^, ^76^ During the preterm period, DTI diffusivities decrease, while anisotropy increases in most WM regions.^85^, ^90^ These DTI parameters continue to show intense changes during the first postnatal months in bundles identified by tractography^67^, ^100^, ^113^ (Fig. 5d). They are probably sensitive to different mechanisms, such as the proliferation of glial cells, the extension of oligodendrocyte processes, and their wrapping around axonal fibers. Maturation models and observations in fetuses, preterm newborns, and infants^61^, ^85^, ^113^, ^114^ supported the hypothesis of sequential changes in DTI parameters and suggested two successive steps: 1) early changes in microstructure related to the fibers premyelination would mainly lead to a decrease in axial and radial diffusivities; and 2) subsequent wrapping of myelin sheaths around axons would not modify axial diffusivity (λ_//_) but would decrease radial diffusivity (λ_┴_), implying an additional increase in anisotropy.

Such a model based on DTI allowed the identification of relevant differences in maturation between WM bundles in infants.^113^ This asynchrony was also observed within a functional network such as the language network: the ventral pathways (uncinate, fronto‐occipital, middle and inferior longitudinal fascicles) appeared more mature than the dorsal ones (arcuate and superior longitudinal fascicles), although this difference decreased during infancy.^115^ From birth to 2 years of age, the trajectories of DTI parameters also showed differences between bundles in terms of asymptote, delay, and speed.^116^ Over this period, the maturational correlation between bundles seems to decrease,^117^ perhaps providing the neural substrates of the asynchronous functional and behavioral acquisitions of infants. Training, as well as experience during development, might influence WM maturation and subsequently alter WM diffusion parameters through increased myelination promoted by neural firing across axons.^118^ Recently, a study evaluating the effect of early music exposure in preterm newborns from 33w PMA to TEA has shown an increase in DTI anisotropy and decrease in radial diffusivity of certain WM fibers.^119^

Beyond DTI, more complex diffusion models have been used to assess WM maturation, such as NODDI, which showed differences between regions in terms of changes in neurite density and orientation dispersion indices in newborns and infants.^79^, ^81^, ^120^ DKI also appeared to be informative in normal development when the estimated intra‐ and extracellular axial diffusivities do not change.^81^ In addition, fixel‐based analysis is a promising quantitative framework to be applied in newborns,^121^ as it allows the separation of fiber populations in voxels containing crossing fibers and it enables fiber cross‐section and density to be characterized. As with the microstructural evaluation of the GM, systematic model‐to‐model comparisons are still lacking to assess which are the best markers to reliably quantify WM maturation in newborns.

#### PREDICTING INFANTS' DEVELOPMENT BASED ON DIFFUSION MRI

In recent years, early diffusion MRI measures have been related to later behavioral development in typical and at‐risk infants, as illustrated in the following examples. Voxelwise analyses of DTI anisotropy highlighted that the WM microstructure in full‐term newborns at ~2 weeks of age is related to neurodevelopmental outcome at ~2 years of age (Bayley scores).^122^ By TEA, the preterm brain has been found to be structurally different from that of healthy full‐term babies. Early alterations of brain networks and their microstructural characteristics were shown to correlate with specific neuropsychological deficits after preterm birth.^123^, ^124^ Such relationships involved several WM and GM regions at TEA, depending on the cognitive, language, and motor scores at 2 years,^125^, ^126^, ^127^ as well as the early structural connectivity between the thalamus and extensive cortical regions.^128^ Using a deep‐learning approach, another study showed that the connectome at birth predicted a 2‐year cognitive score group in both full‐term and preterm infants, the connections involving the frontal lobe being the most important for classification.^129^ These results should be confirmed by other groups, in other cohorts of infants, and with other methodologies, to ensure the clinical relevance.

## Other Quantitative Methods

In addition to previous techniques such as relaxometry and diffusion MRI, other complementary quantitative MRI methods can be used to measure the brain tissue microstructure and maturation in newborns and infants. They are based either on the acquisition of additional sequences or on specific postprocessing tools allowing multiparametric analyses.

### Magnetization Transfer MRI

The magnetization transfer ratio (MTR) informs about the ratio between free water and water with restricted motion, bound to macromolecules such as proteins and lipids. It can be defined as (S_0_ ‐ S_m_)/ S_0_ where S_m_ and S_0_ are the signal intensities measured with and without magnetization transfer (off‐resonance) prepulses, respectively, applied with gradient‐echo or spin‐echo sequences. MTR studies remain rare in the developing brain, perhaps because of limitations related to acquisition time and energy deposition. MTR is thought to reflect the myelin amount as it increases from birth to 2 years of age, at different rates, in the main WM regions and in the central GM nuclei.^130^ However, during the preterm period (26–34w PMA), the corpus callosum has higher MTR values than the posterior limb of the internal capsule,^65^ whereas at this stage callosal fibers are highly organized, closely packed, but nonmyelinated fibers. Between the preterm period and TEA, some regions show increases in MTR while others show decreases (eg, frontal WM including the subplate and intermediate zone).^85^ This technique thus appears to be sensitive not only to myelin‐associated macromolecules, but also to the macromolecular density of axonal cytoskeleton components such as microtubules and neurofilaments.

### Susceptibility‐Weighted MRI

Quantitative susceptibility mapping (QSM) is an emerging technique that measures the magnetic susceptibility (χ_m_) of a tissue and mainly quantifies paramagnetic nonheme iron. Images are acquired with a multigradient echo sequence, and phase images are unwrapped and normalized to different TEs to generate frequency maps. The susceptibility map is computed by removing the background frequency from the average of these maps. QSM appears to provide information on iron, myelin, and macromolecular contents. But as for MTR, there are still few studies available in newborns and infants.

During development, the basic QSM characteristics of brain tissues do not change: GM tends to be paramagnetic (χ_m_ > 0), while WM tends to be diamagnetic (χ_m_ < 0).^131^ In infants, QSM shows age‐related increases in susceptibility in deep GM nuclei, suggesting a gradual iron deposition at different speeds between nuclei.^132^ Besides, the susceptibility values decrease gradually with age in the CSF, and with the myelination degree of WM bundles, following a posterior–anterior spatial and temporal pattern.^131^ QSM has also been used in preterm infants with germinal matrix intraventricular hemorrhage (IVH) in order to better characterize the lesion extent.^133^ At TEA, infants with severe IVH show higher susceptibility values (ie, paramagnetic susceptibility changes) than controls in normal‐appearing WM regions (eg, centrum semiovale, temporal and parietal WM). This is likely related to the accumulation of hemosiderin/ferritin iron secondary to the diffusion of extracellular hemoglobin from the ventricles into the WM.^133^ Susceptibility‐weighted MRI has also been used to characterize punctate WM lesions and cerebellar hemorrhages in infants.^134^, ^135^, ^136^, ^137^

### Perfusion MRI

The developing brain is highly vulnerable to disturbances in blood flow or oxygen supply to cerebral tissues. Advances in MRI have brought forward techniques that allow noninvasive evaluation of brain hemodynamics. Phase‐contrast magnetic resonance angiography (PC‐MRA) and arterial spin labeling (ASL) have been used in the neonatal population (eg, Refs. ^138^, ^139^).

The main advantage of PC‐MRA as compared with ASL is the time of image acquisition. The scan lasts less than a minute to measure volume flow in cm/s, positioned at the basis of the skull. However, it lacks spatial information, and the segmentation of anatomical brain images is necessary to calculate a perfusion value in mL/100 g/min. Quantitative flow volume values are calculated in each vessel by integrating values across manually drawn regions of interest (ROIs) that enclose the vessel lumen of the internal carotid arteries and the basil artery. Both values are further summed and divided by total brain volume (derived from anatomical images) to calculate the total cerebral blood flow.^138^

On the contrary, ASL provides perfusion measurements at the brain tissue level with a subtraction technique between control and labeled images. Arterial hydrogen protons are inverted in the neck region, and a labeled image is acquired after a certain time delay allowing the labeled spins to reach the brain tissue. The perfusion‐weighted image is obtained by subtracting the labeled image and a control image. ASL is limited in SNR, so this label‐control scheme is repeated multiple times to compute the perfusion map (acquisition in about 3 minutes in newborns).^139^ The measures depend on the acquisition settings (eg, label duration, postlabel delay) and patient characteristics (ie, longitudinal relaxation rate or spin–lattice relaxation time of blood, which depends on age and hematocrit, blood flow velocity in the neck). The label efficiency is more likely to vary in neonates. Several technical difficulties have limited the application of ASL in the neonatal population: eg, lower cerebral blood flow (CBF), resulting in lower SNR, and longer tracer lifetime causing negative perfusion. A pseudocontinuous ASL (pCASL) protocol was recently adapted to slow blood flow of preterm neonates.^140^ ASL acquisitions are very sensitive for motion artifacts, which leads to a high rejection rate in the analyses.^141^

**FIGURE 8:**
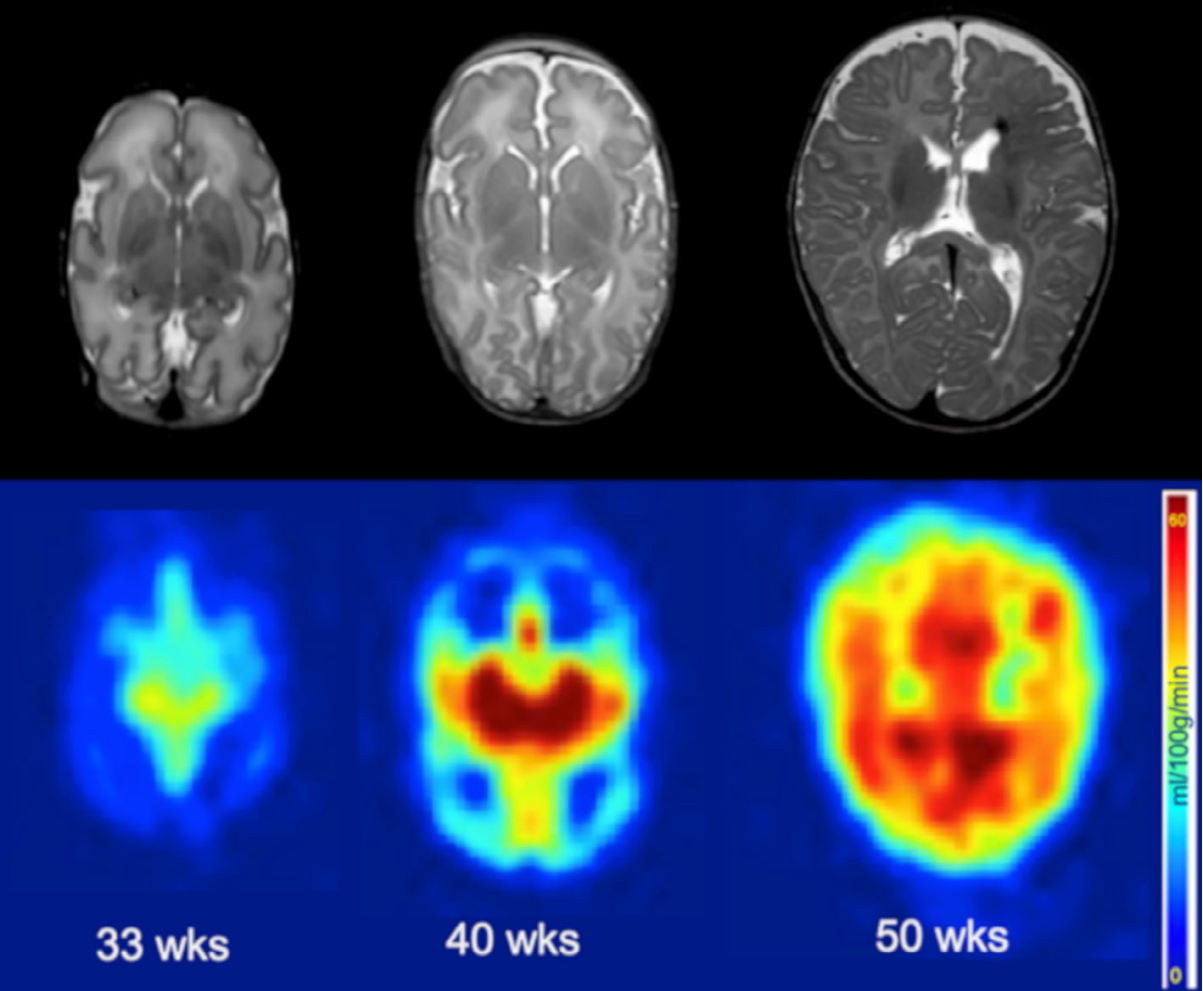
Evolution of brain perfusion during development. Anatomical T_2_w images (top row) and ASL maps (bottom row) are presented for three preterm infants at 33, 40, and 55w PMA, showing the increase in perfusion from deep GM nuclei to the periphery, in occipital regions before frontal regions. Adapted from Ref. ^139^.

Recent studies have observed an increase in CBF with the infants' age, reflecting brain maturation.^140^, ^142^, ^143^, ^144^, ^145^ CBF is the highest in deep GM nuclei, in central and occipital regions compared with frontal regions^139^, ^143^ (Fig. 8), which in turn show the highest rate of CBF increase with age.^140^ In preterm infants at TEA compared with full‐term newborns, differences in brain perfusion have been observed in the basal ganglia in relation with neuromotor outcome.^146^ Lower relative CBF values have also been reported in the insula, anterior cingulate, and auditory regions, and the presence of parenchymal brain injury correlated with lower global and regional CBF.^147^

### Spectroscopy and Chemical Shift Imaging

Proton magnetic resonance spectroscopy (MRS) provides quantification of metabolites based on their different resonant frequency or chemical shift. MRS spectra represents metabolites peaks on a ppm scale. A single‐voxel approach is more robust than multivoxel techniques (ie, chemical shift imaging [CSI]) with better magnetic field homogeneity, higher SNR, resulting in better spectral resolution, less chemical shift or voxel bleeding artifacts, and less contamination from unsuppressed water or lipid signal. Higher field strength allows less spectral overlap and the use of a smaller voxel size, as long as B_0_ homogeneity is optimized. At 3T, an adiabatic selective refocusing sequence is recommended, as well as a 1.5 cm^3^ voxel with 128 averages in neonates.^148^

Changes in metabolites concentration are important during brain development, especially during the first postnatal year, with a rapid increase in N‐Acetyl Aspartate (NAA, peak at 2.02 ppm) and creatine (Cr, peak at 3.03 ppm), and a rapid decrease in choline (Cho, peak at 3.2 ppm) and myo‐inositol (mI, peak at 3.56 ppm).^149^, ^150^ NAA is involved in myelination: it is transported out of the neurons to the oligodendrocytes, where it is used for myelin synthesis.^151^ Creatine is necessary for the regulation of energy supply in cells and is considered to correlate with neuronal cell mass. Total choline is a marker of membrane turnover and myo‐inositol is a glial marker. Both show high values in newborns. Glutamate‐glutamine (Glx, peak at 2.1–2.5 ppm) are used as a marker of the destructive neuronal process. During development, it is involved in different stages of neurogenesis and maturation, including neural progenitor proliferation, migration, differentiation, survival, and synaptogenesis.^151^ Lactate (peak at 1.32 ppm) is a marker of hypoxia or cellular energy failure, but a minimal lactate peak is a normal finding in neonates.^149^

In addition to age changes, metabolites concentration depends on brain location, with higher NAA and Cho values and lower Cr, mI, and Glx values in WM than in GM, and the highest values of Cr and Cho in the cerebellum. Normative curves according to age and brain location can be found in the literature but depend on the acquisition protocol.^151^

In neonates born prematurely, higher concentrations of NAA, Glx, Cr, and mI have been demonstrated in comparison with fetuses of the same age.^152^ At TEA, preterm infants without major WM injuries showed lower levels of Cr, Glx, and macromolecules in the WM, suggesting altered metabolism and protein synthesis,^153^ lower mI concentration suggesting possible astrogliosis,^154^ lower NAA/Cho ratio in the thalamus in association with neurodevelopmental delay at 18 months of age,^155^ and lower NAA and higher Cho levels in the cerebellum.^156^ The presence of cerebellar injury was consistently associated with reduced concentrations in NAA, Cho, and Cr.^156^ Preterm infants with punctuate WM lesions also showed a reduced NAA level in the parietal WM, suggesting neuronal damage and myelin injury.^154^

### Other Recent MRI Approaches

Different methods are therefore available to quantitatively assess the brain maturation of newborns. But to date, very few studies have actually compared the available markers and evaluated their complementarity or redundancy. In recent years, some sophisticated approaches have been proposed to characterize maturation mechanisms based on previous quantitative MRI methods. Different teams have implemented multicompartmental or multiparametric methods to compare or integrate complementary parameters.

#### Complex markers of maturation

One of these recent approaches aimed to provide a quantitative marker of the amount of myelin in the developing brain by computing the volume fraction of water related to myelin (f_my_). This relies on a multicompartmental model estimated based on the distributions of T_1_ and/or T_2_ relaxation times in each voxel of the tissues, which requires measuring MR signals for different acquisition settings (eg, different TEs, different TIs). Different methods for data acquisition and analysis have been proposed to estimate the parameters of interest (eg, f_my_), with variable reliability depending on the model assumptions (eg, the compartment relaxation characteristics). The method measuring f_my_ based on the imaging technique mcDESPOT (multicomponent driven equilibrium single pulse observation of T_1_/T_2_) has provided a spatiotemporal pattern in line with prior histological studies of myelination during infancy and toddlerhood, with the maturation beginning in central brain regions and proceeding in a caudocranial way.^157^ A data‐driven technique (ie, independent component analysis [ICA]) has further enabled parcellating WM regions according to their f_my_ trajectories.^158^ The trajectories of some regions highlighted in this way were associated with individual differences in cognitive abilities.

**FIGURE 9:**
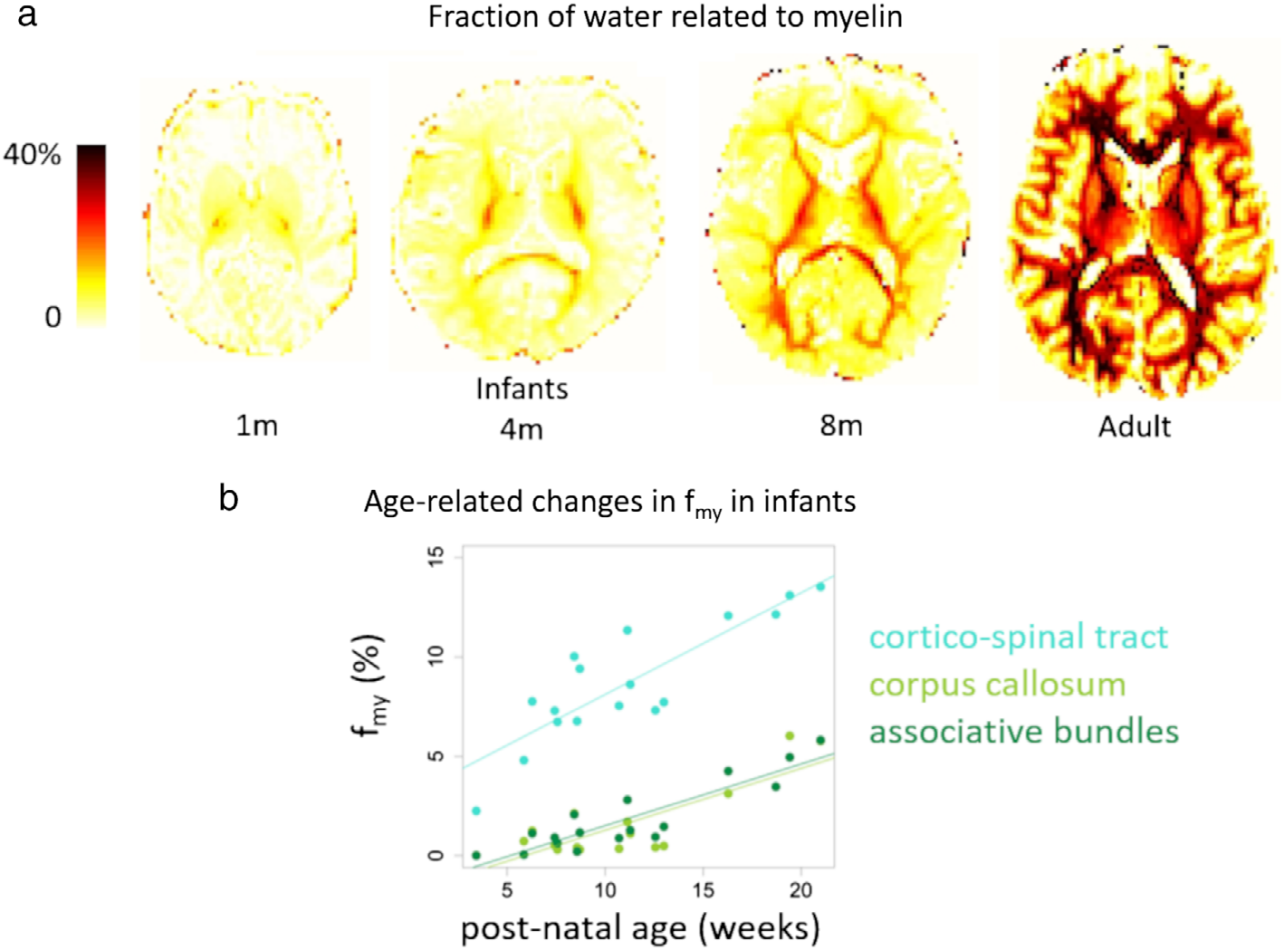
The evolution of the fraction of water related to myelin during development. The f_my_ maps are presented for term‐born infants at 1, 4, and 8 months of age and an adult (same subjects as in Fig. 2), showing the progression of myelination from central regions to the periphery **(a)**. This fraction increases with age in the WM, at different rates between bundles **(b)**. Adapted from Refs. ^16^, ^159^.

The main drawback of this method is the long acquisition and/or postprocessing times; an alternative method based on EPI sequences was proposed, with a precalibration step of the model parameters, performed on a few adults.^159^ The whole‐brain maps obtained in infants with a 5‐minute acquisition protocol showed the progression of myelination from central to peripheric regions (Fig. 9a). In the BCP project, a 2D MR fingerprinting method has been developed to measure f_my_ simultaneously with T_1_ and T_2_ relaxation times.^70^ The first results showed a negligible level of f_my_ until 6 months of age and a gradual increase afterwards, with regional differences (Fig. 9b). Compared with T_1_ and T_2_ parameters, f_my_ seems to provide differential but complementary sensitivity to the tissue maturation changes.^70^, ^157^

Other recent composite methods proposed to estimate the so called “g‐ratio” (ie, the ratio of the axon diameter to the outer fiber diameter including the myelin sheath), based on diffusion‐ and myelin‐related measures (using, for instance, NODDI indices, MTR, or f_my_ measures). This marker is expected to decrease in the WM with the myelination process, as it has been observed in premature infants imaged at 30 and 40w PMA^160^ and in young children.^161^ This index might provide relevant information on the efficiency of neural information transfer and on the conduction velocity of WM pathways. However, the complementarity of these different markers, in relation with maturation mechanisms, remains to be assessed over different developmental periods.

#### COMPARISONS OF PARAMETERS

A few studies have begun to explore this issue. The comparison of T_1_, MTR, and DTI parameters in infants scanned during the preterm period (28–32w PMA) and at TEA showed that these maps have distinct contrasts, with differences between brain regions and across ages.^65^ Unlike the other parameters, the MTR pattern of regional variation changed between the preterm age and TEA. Voxel‐based analyses and comparison of these parameters further highlighted the lamination pattern in the cerebral wall and suggested different maturation mechanisms in the brain compartments (eg, subplate, intermediate zone).^85^ In preterm infants between 27 and 58w PMA, NODDI, T_2_ and f_my_ properties were also compared in different brain regions.^162^ This study showed that, in the thalamus, diffusion age‐related changes are not solely due to myelination, while diffusion and T_2_ changes are related in the posterior WM (probably due to axonal and glial proliferation) but do not rely on the myelin water content.

#### MULTIPARAMETRIC APPROACHES

**FIGURE 10:**
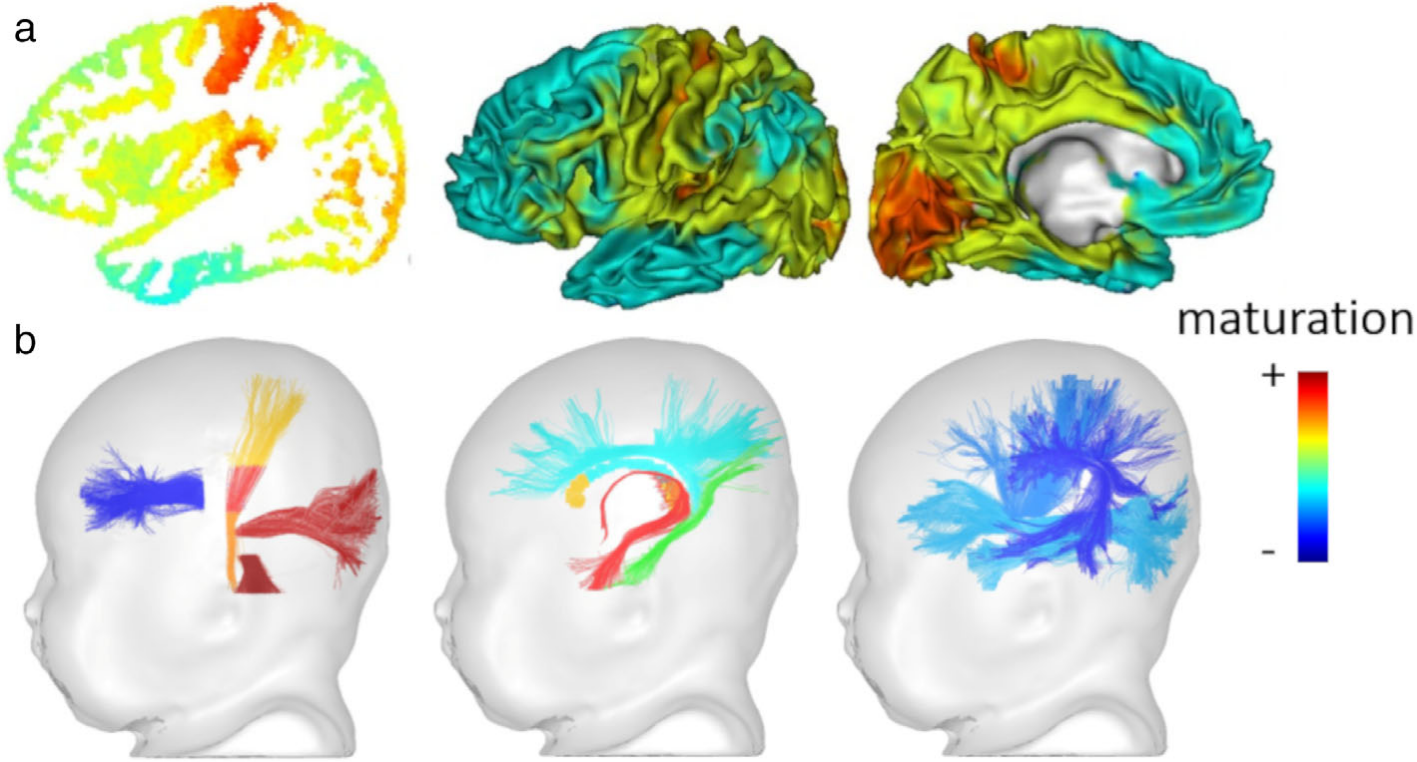
Asynchronous maturation of brain networks. Based on multiparametric MRI approaches, heterogeneities in maturation are highlighted across cortical regions (**a**: method using quantitative T_1_ and DTI axial diffusivity; adapted from Ref. ^68^) and WM bundles (**b**: method using quantitative T_1_ and T_2_, DTI axial and radial diffusivities; adapted from Refs. ^4^, ^67^).

In recent years, other approaches have proposed to integrate the complementary information provided by MRI parameters (T_1_ and T_2_ relaxation times, anisotropy, and diffusivities from DTI) in infants between 1 and 5 months of postnatal age, with the aim to consider jointly changes related to various maturation mechanisms (eg, changes in cell and membrane density, in water and iron content, in relation with the development of dendritic arborization, synaptogenesis, fiber myelination, etc.). First, an approach was considered to group brain voxels based on their similar properties, with a clustering algorithm applied to combinations of these indices.^68^, ^69^ This allowed the classification of cortical regions according to their maturation, without any hypothesis *a priori* on their anatomical location.^68^ The resulting maps showed the different maturation patterns of cortical regions at the individual level, as well as the progression over the infant group according to age. This confirmed the early maturation of primary sensorimotor regions, followed by adjacent unimodal associative regions, and finally by higher‐order associative regions (Fig. 10a). T_1_ and DTI axial diffusivity (λ_//_) then seemed to be the most relevant parameters to evaluate the GM characteristics over this developmental period. The same approach applied to WM voxels highlighted how the maturation proceeds from the brain center to the periphery.^69^

In addition, a maturation distance (based on the Mahalanobis distance) was estimated in a set of WM bundles, by comparing T_1_, T_2_, and DTI diffusivities in infants compared to a group of adults.^67^ This revealed more maturational relationships between bundles than univariate approaches, and it allowed to quantify their relative delays of maturation. The results confirmed the intense changes during the first postnatal year, as well as the maturational asynchrony across bundles, notably with early maturation of the spinothalamic tract, optic radiations, corticospinal tract and fornix, and delayed maturation of associative bundles such as the superior longitudinal and arcuate fasciculi (Fig. 10b).

Overall, these recent research studies have provided interesting tools and microstructural markers that might better reflect the complexity and overlapping patterns of maturation mechanisms in the GM and WM tissues throughout development. But much work remains to be done to ensure the translation of these methodologies into the clinical context.

## Functional MRI

During early infancy, structural maturation mechanisms, measured with the MRI techniques described previously, are accompanied by the functional development of the brain, allowing infants to acquire sensorimotor and cognitive capacities according to their perceptions of the environment, experiences, and learning. Indeed, although immature at birth, the brain shows an early architecture.^23^ Nevertheless, important plasticity phenomena can come into play and modify the early established architecture, particularly under the constraint of various disturbances (eg, perinatal lesion).

### Developmental Specificity and Methodological Challenges of fMRI

The method most commonly used in adults to map the brain functional networks is fMRI. Nevertheless, it remains challenging in infants.^163^ As with the previous MRI techniques, short protocols might be preferred to target moments of calm for the baby. Indeed, head movements are a main issue, also because this behavior might have different causes and vary between groups of infants (typical or at risk for atypical development), leading to “artifactual” confounds in fMRI studies.^164^ In recent years a custom‐designed MR coil has been proposed to optimize the temporal SNR according to the newborn head size^165^ and the dHCP project uses a custom‐designed 32‐channel neonatal‐specific head coil.^28^ Maximizing the sensitivity to the BOLD (blood oxygen level‐dependent) brain activity also requires using optimal TE in relation to the T_2_* characteristics of the developing brain (longer T_2_* in newborns and infants than adults, because of the higher water concentration and lower lipid content). For 3T fMRI of full‐term newborns, a TE of ~50 msec has been recommended to detect significant stimulation‐related BOLD changes.^166^

Regarding the postprocessing of fMRI data, several steps are to be considered in order to maximize the reliability and precision of functional activation maps. Recently, the dHCP resting‐state fMRI (rs‐fMRI) preprocessing pipeline has been extended for the analysis of stimulus–response fMRI images.^167^ Implementing optimized motion and distortion corrections, ICA‐based denoising, and hemodynamic modeling led to a substantial increase in the spatial specificity and sensitivity of functional maps.^167^ Indeed, the BOLD response characteristics (ie, time‐course, amplitude) evolve during development. Measuring an accurate model of the hemodynamic response function (HRF) in the targeted population of subjects is thus required. For example, with a passive motor stimulation paradigm and an event‐related design, the HRF waveform has been characterized in preterm newborns at 32–35w PMA, at TEA (38–44w PMA), and in adults, and a systematic maturational trend was observed: the amplitude of the main positive peak increased with age while its time decreased.^168^

Contrary to adults, BOLD responses with either positive or negative main peaks have been reported in neonates and infants,^169^ which has led to a controversy on the developmental changes in CBF and oxygen consumption following neural activity. Indeed the neurovascular coupling (mechanism linking the transient neural activity to the subsequent change in blood flow), as well as the autoregulatory system, seem to differ in the developing brain compared to adults.^170^ In a systematic study in neonatal rats, the developmental evolution of cortical blood flow has been characterized.^171^ P12 rats (equivalent to human newborns) exhibited an “inverted” hemodynamic response (~negative BOLD) with early oxygen consumption followed by delayed constriction of pial arteries. These responses varied with the stimulus‐evoked systemic blood pressure, leading to cortical hyperemia (~positive BOLD) in some cases. In older rats, the hemodynamic response matured with the development of an initial hyperemic phase (~positive BOLD) that eventually masked oxygen consumption and balanced vasoconstriction toward adulthood.^171^ Such dynamical changes might explain the variability in BOLD responses described in previous fMRI studies of newborns and infants.

### Mapping Functional Networks with “Task‐Based” fMRI

In recent years, fMRI during stimulation paradigms has been used to explore how the developing brain processes sensorimotor and cognitive information (eg, somatosensory, visual, language stimuli).

#### SENSORIMOTOR SYSTEM

**FIGURE 11:**
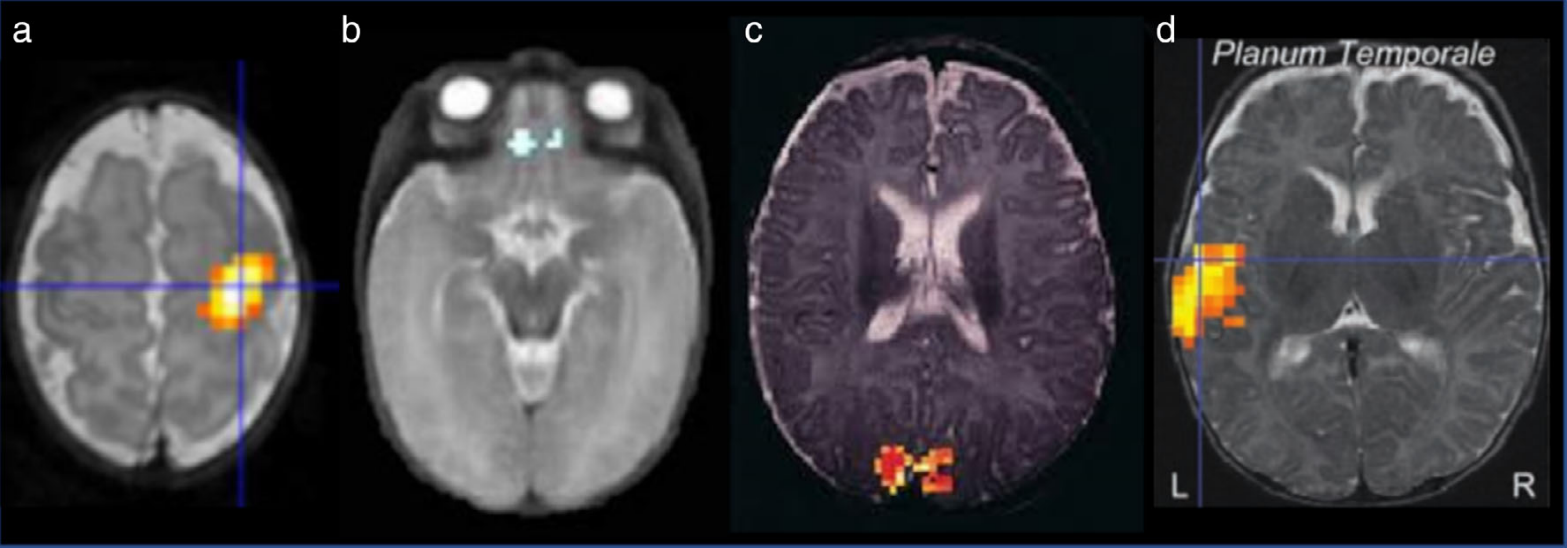
Functional mapping in newborns and infants. Functional MRI studies have described the brain regions activated in groups of newborns and infants following sensorimotor stimulations (**a:** adapted from Ref. ^165^), olfactive stimulations (**b:** adapted from Ref. ^172^), visual stimuli (**c**. adapted from Ref. ^173^, and speech stimuli (**d:** adapted from Ref. ^174^). It should be noted that the brain regions involved are very similar to those of adults, suggesting an early organization of functional networks despite their low maturation.

Somatosensory perceptions and motor activities are among the first that fetuses experiment inside the womb. The sensorimotor network has been studied based on passive motor or tactile stimulation paradigms (Fig. 11a). Brain responses following induced wrist movements have been identified in the contralateral primary somatosensory and motor cortices in preterm newborns as young as 30w PMA.^175^ The network involved seems to expand and specify during development: whereas more spatially dispersed responses and progressive integration of sensorimotor associative areas and ipsilateral hemisphere were first observed until TEA, an age‐related increase in spatial specificity has been highlighted in older infants.^175^ FMRI also showed that a rough somatotopic organization of the primary sensorimotor cortices is in place from 32w PMA, with different brain regions activated by stimulation of the wrists, ankles, and mouth.^176^ To investigate how the developing brain processes affective touch, another team measured brain responses to gentle skin stroking in full‐term newborns under 1 month of age, and showed activations in two regions also involved in the mature brain (postcentral gyrus and posterior insular cortex).^177^ These studies suggest an early functional organization of the sensorimotor network from the preterm period.

#### SENSE OF SMELL SYSTEM

Smell is the second sense to develop in fetuses *in utero*. After birth, it is expected to play a crucial role for behavioral adaptation, interactions, and bonding processes between the newborn and mother. A recent fMRI study in newborns has shown that adult‐like cortical regions (including piriform cortex, orbitofrontal cortex, and insula) are activated by the perception of olfactory and trigeminal odorants^172^ (Fig. 11b), suggesting an early specialization of the smell brain network.

#### VISUAL SYSTEM

Vision is also one of the first sensory functions to develop in humans. Visual stimulations are quite limited *in utero*, but spontaneous retinal activity allows the visual pathways to begin to function and specialize early in the third trimester of pregnancy. Birth triggers an avalanche of visual stimuli that will induce increased cerebral activity and a cascade of maturation mechanisms. fMRI visual protocols are difficult to implement in infants, so the first studies were conducted in sedated subjects and revealed negative and then positive BOLD responses in newborns and infants (Fig. 11c).^169^ Recently, however, two groups have been successful in studying awake infants. Based on flow vs. random‐motion stimuli, the major cortical regions responsible for visual motion perception were shown to be active in 2‐month old infants, and independent visual inputs to primary (V1) and temporo‐occipital (V5/MT+) regions seem to exist early on.^178^ Despite differences in response profiles and activity patterns, the extrastriate visual cortex was shown to present an early adult‐like specialization and a spatial organization for visual categories (eg, faces, scenes) in 4–6‐month‐old infants.^179^ These studies suggest that a few months after birth, the developing brain is already capable of high‐level visual processing, although the underlying network is further refined during childhood.

#### AUDITORY/LANGUAGE SYSTEMS

The maturation of the auditory system extends over a longer period than that of the visual system, from pregnancy to childhood. The brain network dedicated to auditory and language processing has been studied by different groups with fMRI. In 3‐month‐old infants, speech stimuli already evoke activity in left‐lateralized brain regions (including the superior temporal and angular gyri)^174^ (Fig. 11d). As in adults, perisylvian regions show different speeds of activation (with fastest responses close to Heschl's gyrus) and different sensitivities to sentence repetition (with increasing activity in inferior frontal Broca's regions, suggesting early involvement in verbal memory).^180^ In comparison with biological nonspeech sounds, the temporal region becomes increasingly selective for speech between 1 and 4 months of age.^181^ On the other hand, in full‐term newborns, music listening evokes right‐hemispheric activations of primary and higher‐order auditory cortical regions.^182^ Compared with full‐term newborns, preterm infants at TEA (38–41w PMA) show less posterior thalamic activations to linguistic stimuli but similar bilateral activations in superior temporal, supramarginal, and inferior frontal gyri.^183^ At an early age (29–34w PMA), superior temporal and supramarginal activations have also been observed, with a left prevalence, while at a later age (44–45w PMA) the pattern of activity seems to refine, with bilateral superior temporal and left‐lateralized supramarginal activations. From TEA, these brain responses seem partly related to the neuropsychological outcome of preterm infants.^183^ As a whole, these studies suggest an early lateralized specialization for speech and music processing, which would be modulated by the experienced environment during the preterm period.

The advances made possible by these task‐based fMRI studies in newborns are fundamental. Nevertheless, they remain difficult to implement, especially since the conditions of installation in the MRI scanner, the noise of the acquisitions, and the sensitivity to movements reduce the possibility that the baby is awake and calm during the protocol. The potential of these studies in a clinical context therefore seems to be reduced without major advances in these different domains.

### Mapping Resting‐State Networks

**FIGURE 12:**
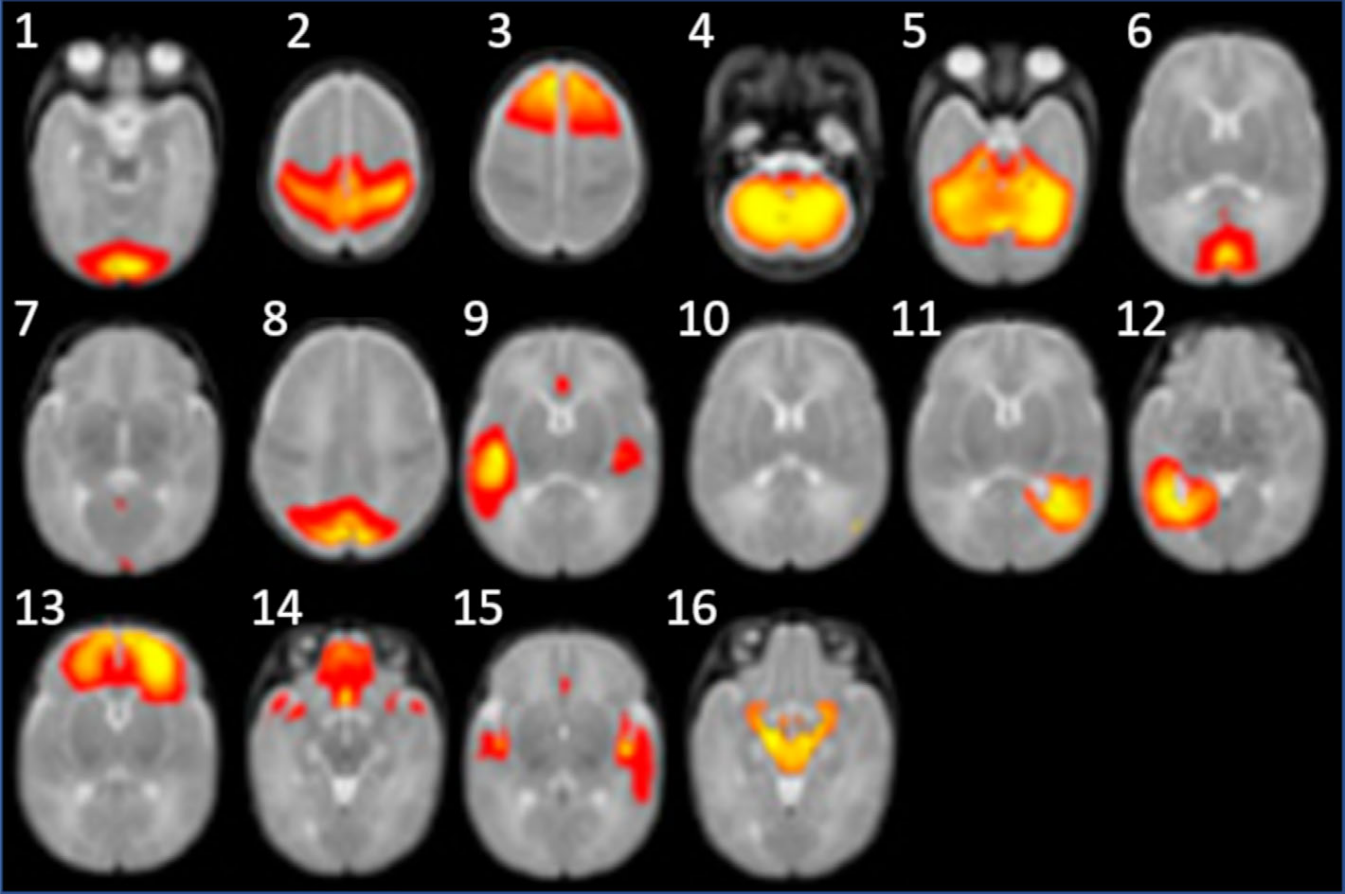
Resting‐state fMRI mapping in newborns. Components obtained from ICA analysis in full‐term newborns and preterm infants at TEA are presented on axial view (threshold at z‐score >3) and superimposed on a T_2_‐weighted MR infant brain template. The highlighted networks include visual (1), sensorimotor (2), superior frontal regions (3, 13), cerebellum (4), posterior cingulate cortex (6), precuneus (8), auditory (9), posterior temporal (11,12), orbitofrontal regions (14), the salience network (15), brainstem and thalami (16), as well as noise components (5, 7, 10). Adapted from Ref. ^187^.

Another promising technique to study the development of functional networks in newborns is rs‐fMRI. It allows mapping networks of brain regions that are “functionally connected,” ie, regions showing spontaneous and coherent BOLD signal fluctuations over time. Usually, fluctuations at low frequencies (eg, at 0.01–0.08 Hz corresponding to time periods of 12.5–100 sec) are targeted. However, analyses of functional connectivity in high‐frequency bands have also been proposed, as well as ones of dynamic (instead of stationary) functional connectivity.^184^ The main advantage of rs‐fMRI in newborns is that data are acquired in the absence of any task. But it implies several methodological challenges, as detailed in recent reviews.^184^, ^185^, ^186^ With regard to data analyses, a first method is ROI‐based and provides voxelwise correlation analysis on the whole brain. The results are easily interpretable, but they are also highly dependent on the ROIs anatomical definition. Identifying individual regions based on the propagation of a parcellation atlas is possible. Nevertheless, this requires an age‐matched atlas, as well as accurate registration within the group.^184^ Another approach relies on data‐driven ICA (Fig. 12), which allows the exclusion of noise components in further analyses.

Recent studies have shown that the newborn brain demonstrates a complex resting‐state functional architecture, with developmental changes such as an increase in interhemispheric connectivity during the preterm period.^107^ Analysis of connectome gradients in full‐term newborns has revealed differences between unimodal and transmodal regions, suggesting an adult‐like organization under construction.^188^ Networks located in visual, sensorimotor, and auditory regions are already present shortly after birth,^189^ but their volume and strength of activation increase during the first 2 years of age.^190^ Between 4 and 9 months of age, a similar network localization is observed but the connectivity strength decreases in local networks and increases in more distant networks.^191^ Regarding higher‐order cognitive processes, an immature default mode network (“proto‐DMN”) is usually found in newborns, whereas integrated network structures more similar to adult DMN (relying on ventromedial prefrontal, posterior cingulate, medial temporal regions, precuneus, and angular gyrus) appear at 1 year of age.^192^, ^193^, ^194^, ^195^ Central executive network (relying on dorsolateral prefrontal, posterior cingulate, and posterior parietal regions) is also observed in full‐term newborns,^189^ although connectivity within the network increases during development. As for the salience network (relying on anterior insula and dorsal anterior cingulate region), local specialization and connectivity strength dramatically increase during the first 2 years of age.^196^

Some studies in newborns have begun to compare the developing functional and structural connectivity patterns, as provided by rs‐fMRI and diffusion MRI. The mechanisms linking these measures are still poorly understood in adults: the strength of structural connections seem to predict the strength of functional connections, whereas strong functional connections exist between regions with no direct structural connections.^197^ Some groups have suggested that structural approaches could primarily reflect monosynaptic connections, while functional approaches could also be sensitive to polysynaptic connections.^197^ Studies in newborns have also revealed that the similarities/dissimilarities between the structural and functional connectivity patterns depend on the systems, with greater overlap in sensory regions and pathways than in higher‐order association ones.^107^, ^188^, ^198^ This might be due to differences in maturation between systems, leading to technical biases such as more reliable tractography reconstructions of mature structural connections (eg, sensorimotor compared to associative ones).^98^ On the other hand, associative regions might show delayed functional integration despite already established structural connections.

Prematurity impacts the functional connectivity between resting‐state networks. Compared with full‐term newborns, preterm infants at TEA show less complex intrinsic activity, less robust interhemispheric and thalamocortical connectivity, and less connectivity between thalamus and the salience network.^199^, ^200^, ^201^, ^202^ They also show reduced coupling in a circuit of three network modules interconnected by the salience network.^187^ These modules consisted of 1) medial superior frontal, auditory, and sensorimotor networks; 2) orbitofrontal, posterior cingulate regions, precuneus, visual and left posterior temporal networks; and 3) thalamus, precuneus, and right posterior temporal networks. Environmental enrichment through music exposition improved the functional connectivity in this particular circuit.^187^ This study also proposed an original approach to analyze functional connectivity based on nonparametric estimators of accordance and discordance to increase the sensitivity and robustness of detecting group effects. It assumed that extreme events of the BOLD fluctuations represent significant activations or deactivations of the ROI, while spurious fluctuations are considered noise.

Overall, the rs‐fMRI approach holds promise for understanding the establishment of functional networks in newborns, especially when the results can be compared with those provided by complementary MRI methods for investigating structural maturation.

## Conclusion and Perspectives

In recent years, a wide variety of novel methodologies have been proposed and implemented to allow the MRI study of the developing brain *in vivo* in newborns and infants. These varied approaches allow the increasingly precise exploration of multiple neurodevelopmental mechanisms, from morphological to microstructural changes observed in both GM and WM, in addition to metabolic and functional changes. Nevertheless, studies of the developing brain remain limited in several respects, given the intrinsic methodological and experimental issues. Providing general recommendations is difficult without knowing the question being asked, the clinical or research context, and the available acquisition time for protocol in newborns. Quality control procedures are essential to ensure the biological veracity of MRI observations. Hence, many challenges remain to be met in order to study, in the most automatic and reliable way possible, cohorts with a large number of subjects and different ages, or groups of newborns with different early pathologies. High‐field MRI (ie, 7T MRI) would also be an exciting and promising perspective for mapping, with high spatial resolution, fine structures in development, or microlesions not visible with 3T MRI. This is a hot topic that some collaborative research and clinical groups have begun to address, but which triggers additional constraints and challenges.

Another essential perspective currently concerns multimodal approaches, aiming to combine brain MRI measures with clinical and behavioral markers, or electrophysiological indices such as those provided by electroencephalography (EEG).^98^ For instance, an increased activity in the first postnatal days in preterm newborns (as measured with EEG signal peak‐to‐peak amplitude and “spontaneous activity transients”) has been related to a faster growth of the brain and subcortical GM during subsequent weeks until TEA.^203^ In typical infants, research studies have further suggested important links between MRI microstructural indices of WM maturation and EEG functional measures (ie, response latency and speed) for the visual system.^204^, ^205^ But such relationships seem less clear for the auditory system,^93^ suggesting more complex interaction mechanisms, in which the environment, for example, could play a more prominent role.

Understanding such developmental asynchronies with regard to the early sensory and integration capacities of infants remains an exciting question to explore. MRI has a key role to play here, as well as to better characterize and diagnose neurodevelopmental pathologies from pre‐ or perinatal origins. Since it can be achieved well before knowing the child's behavioral and clinical outcome, MRI is an unavoidable exploration method to evaluate the efficiency of early neuroprotective or neuroregenerative interventions, or remediation strategies aiming to avoid long‐term disabilities of children.
